# Radiomics-Based AI for Predicting Neoadjuvant Immunochemotherapy Pathological Response in Non–Small Cell Lung Cancer: Systematic Review and Meta-Analysis

**DOI:** 10.2196/93892

**Published:** 2026-08-13

**Authors:** Ziqi Jiang, Yuan Xu, Shuyu Jia, Hongsheng Liu

**Affiliations:** 1 Department of Thoracic Surgery, Peking Union Medical College Hospital, Chinese Academy of Medical Science, No.1 Shuaifuyuan, Wangfujing, Beijing, Dongcheng District, 100730, China, 86 13621021237; 2 Department of Infectious Diseases, Nanfang Hospital, Southern Medical University, Guangzhou, China

**Keywords:** radiomics, non–small cell lung carcinoma, neoadjuvant therapy, pathological response, meta-analysis, NSCLC

## Abstract

**Background:**

Non–small cell lung cancer (NSCLC) remains the leading cause of cancer-related mortality worldwide. Accurate early prediction of response to neoadjuvant therapy is critical.

**Objective:**

We aimed to evaluate the diagnostic performance of radiomics-based AI in predicting pathological complete response (pCR) and major pathological response (MPR) following neoadjuvant immunochemotherapy in NSCLC and to compare it against traditional radiological criteria.

**Methods:**

A systematic search of PubMed, Embase, Cochrane Library, and Web of Science was conducted through October 26, 2025. Studies utilizing computed tomography (CT) or positron emission tomography/CT-based AI models to predict pCR or MPR were included. Methodological quality was appraised using the PROBAST (Prediction Model Risk of Bias Assessment Tool)+AI tool. Sensitivity, specificity, and area under the curve (AUC) were pooled using a bivariate random effects model.

**Results:**

Twenty-three studies involving 2004 patients in validation sets were included, with histopathology as the gold standard. For pCR, AI models achieved a pooled sensitivity of 0.77 (95% CI 0.70‐0.83), specificity of 0.79 (95% CI 0.73‐0.84), and AUC of 0.85, significantly outperforming traditional criteria in sensitivity (0.77 vs 0.42; *P*<.001). For MPR, AI demonstrated a sensitivity of 0.80 (95% CI 0.72‐0.87), specificity of 0.83 (95% CI 0.73‐0.90), and AUC of 0.88, also superior to traditional models (AUC: 0.88 vs 0.65, *P*<.001). Subgroup analysis revealed that positron emission tomography/CT-based models offered higher specificity for MPR than CT-based models (0.95 vs 0.80, *P*=.005).

**Conclusions:**

Radiomics-based AI demonstrates high diagnostic accuracy and superior sensitivity compared to traditional radiological criteria, showing significant potential for preoperative response assessment. However, the heterogeneity and retrospective design of current studies limit the evidence. Future large-scale, prospective, multicenter trials and multimodal data integration are required to validate these findings for clinical translation.

## Introduction

Non–small cell lung cancer (NSCLC) remains the leading cause of cancer-related mortality worldwide, accounting for approximately 85% of all lung cancer cases [1]. While neoadjuvant immunochemotherapy has transformed the therapeutic landscape for resectable NSCLC, patient responses exhibit significant interindividual heterogeneity. Consequently, accurate early prediction of response to neoadjuvant therapy is critical. Precision in early assessment not only optimizes the surgical window but also prevents nonresponders from incurring unnecessary toxicity and delays in accessing alternative therapeutic strategies.

Clinically, major pathological response (MPR) and pathological complete response (pCR) are generally regarded as the gold standards for evaluating the efficacy of neoadjuvant therapy. However, these metrics are characterized by delayed availability (obtainable only postoperatively) and are constrained by the subjectivity of pathological assessment as well as sampling limitations, precluding a comprehensive evaluation of the patient’s systemic tumor status [2,3]. Furthermore, immunotherapy-induced alterations in the tumor microenvironment (such as extensive necrosis and fibrosis) make it challenging to distinguish residual viable tumor from treatment-related reactive changes, rendering the evaluation results highly dependent on experienced pathologists [3]. In preoperative assessment, clinical practice commonly relies on traditional imaging criteria such as Response Evaluation Criteria in Solid Tumors 1.1 (RECIST 1.1) or Positron Emission Tomography Response Criteria in Solid Tumors (PERCIST). Nevertheless, these conventional tools, based on morphological or metabolic features, demonstrate significant limitations within the context of immunotherapy. The anti-tumor immune response itself can increase metabolic uptake in lesions and lymph nodes, resulting in the “flare” phenomenon [4]. Consequently, traditional criteria often fail to accurately differentiate viable tumor from treatment-related changes, leading to an underestimation or misjudgment of therapeutic efficacy.

Recently, radiomics-based AI has emerged as a promising noninvasive tool, capable of decoding high-dimensional quantitative features invisible to the human eye to capture tumor heterogeneity [5,6]. Radiomics extracts a large panel of mathematically defined features—including first-order statistics, shape descriptors, and higher-order texture matrices—from standard-of-care medical images, effectively converting qualitative imaging phenotypes into mineable quantitative data [7]. When coupled with machine learning or deep learning algorithms, these features can model complex, nonlinear relationships between imaging phenotypes and underlying tumor biology, including treatment-induced changes in cellularity, vascularity, and immune infiltration that precede macroscopic anatomical change [8]. In the context of oncologic imaging, radiomics-based AI has been applied across multiple cancer types and imaging modalities—including computed tomography (CT), magnetic resonance imaging, and positron emission tomography (PET)/CT—for tasks ranging from differential diagnosis to prognostication [9]. However, despite this rapidly expanding literature, three persistent barriers impede clinical translation: (1) a lack of standardization in feature extraction, model development, and reporting; (2) insufficient external validation across independent cohorts and institutions; and (3) a paucity of evidence demonstrating clinical utility beyond retrospective discrimination metrics. Within NSCLC specifically, while preliminary studies suggest that AI may offer a superior predictive value for neoadjuvant response assessment, current literature presents inconsistent performance results across different imaging modalities and validation cohorts [10-12]. A critical and unresolved question remains whether these AI models definitively outperform traditional radiological response criteria in clinical utility, necessitating a rigorous systematic evaluation.

Therefore, it is essential to systematically evaluate the performance of radiomics-based AI in predicting MPR and pCR to neoadjuvant immunochemotherapy in NSCLC. Furthermore, this study aims to benchmark AI performance against traditional radiological response criteria and investigate potential covariates influencing diagnostic accuracy, thereby providing high-level evidence for clinical translation. From a digital health perspective, establishing rigorous evidence on the diagnostic performance of these AI models is a prerequisite for their integration into digital clinical workflows, including preoperative decision support systems, multidisciplinary virtual tumor boards, and teleconsultation platforms, thereby advancing the translation of AI-enabled imaging biomarkers into routine digital oncology practice.

## Methods

### Protocol and Guidelines

This systematic review and meta-analysis was conducted in strict adherence to the PRISMA-DTA (Preferred Reporting Items for Systematic Reviews and Meta-Analyses for Diagnostic Test Accuracy) guidelines [13]. The completed PRISMA checklist is provided in Checklist 1. The protocol for this study was prospectively registered in PROSPERO (International Prospective Register of Systematic Reviews; CRD420251243962).

### Search Strategy

A comprehensive literature search was conducted across PubMed, Embase, Cochrane Library, and Web of Science databases, with the final search executed on October 26, 2025. The search strategy was constructed using Boolean operators (AND/OR) combining MeSH and free-text terms across three conceptual blocks: (1) AI terms (“artificial intelligence,” “machine learning,” “deep learning,” “radiomics,” “neural network”); (2) NSCLC terms (“non-small cell lung cancer,” “NSCLC,” “lung neoplasms”); and (3) neoadjuvant immunochemotherapy terms (“neoadjuvant therapy,” “immunotherapy,” “chemotherapy,” “immune checkpoint inhibitor”). Database-specific syntax was applied: PubMed employed [MeSH] tags, title/abstract field restrictions [tiab], and truncation (*); Embase utilized/exp (Emtree) and :ti,ab,kw field qualifiers; Web of Science employed TS=field tags; and Cochrane Library used :ti,ab,kw qualifiers within the Cochrane Reviews and Trials registers. No language or publication date filters were applied during the search phase to maximize sensitivity. The complete, reproducible search strings for each database, including exact syntax, are provided in Table S1 in Multimedia Appendix 1. Two independent reviewers (ZJ and YX) systematically screened retrieved records by title and abstract, followed by a rigorous full-text assessment. To ensure exhaustiveness, the reference lists of all included studies were manually scrutinized to identify potential additional citations.

### Inclusion and Exclusion Criteria

The inclusion criteria were strictly defined using the PITROS framework:

- Participants (P): patients with pathologically confirmed NSCLC undergoing neoadjuvant immunochemotherapy followed by radical resection
- Index tests (I): AI models (machine learning and deep learning) derived from CT or PET/CT imaging
- Target conditions (T): MPR (≤10% residual viable tumor) and pCR (0% viable tumor)
- Reference standard (R): standardized histopathological assessment of surgical specimens
- Outcomes (O): predictive performance metrics, sensitivity, specificity, and area under the curve (AUC)
- Setting (S): retrospective or prospective cohorts from single-center, multicenter, or public databases

Conversely, studies were systematically excluded based on the following criteria: (1) inappropriate publication types, including reviews, case reports, conference abstracts, meta-analyses, and letters; (2) lack of relevance to the specific target conditions (MPR or pCR) or absence of radiomics-based AI models; and (3) insufficient data to reconstruct 2×2 contingency tables. The screening process was conducted independently by 2 reviewers (ZJ and YX), with any discrepancies resolved through consultation with a third adjudicator (HL) to ensure rigorous selection.

### Quality Assessment and Certainty of Evidence

Two reviewers (YX and ZJ) independently assessed the risk of bias using the PROBAST (Prediction model Risk of Bias Assessment Tool)+AI tool [14]. The comprehensive set of signaling questions and the resulting assessment matrices are documented in Tables S2 and S3 in Multimedia Appendix 1. The certainty of evidence was evaluated using the GRADE (Grading of Recommendations Assessment, Development and Evaluation) framework [15], covering risk of bias, indirectness, inconsistency, imprecision, and publication bias (Table S4 in Multimedia Appendix 1).

### Data Extraction

Data were independently extracted by 2 reviewers (ZJ and YX), with discrepancies resolved by a third (HL). Extracted variables included patient characteristics, imaging parameters, and AI performance metrics. When diagnostic contingency tables were not directly reported, values for true positives (TP), false positives (FP), false negatives (FN), and true negatives (TN) were reconstructed using the reported sensitivity, specificity, total sample size (N), and the number of patients with the target condition as determined by the gold standard histopathological assessment (D+). For each study, N_non-disease=N − D+, and the 2×2 cell counts were back-calculated as follows: TP = sensitivity × D+, FN = D+ − TP, TN = specificity × (N − D+), and FP = (N − D+) − TN. All reconstructed counts were rounded to the nearest integer. As a verification step, sensitivity and specificity were recalculated from the derived TP, FP, FN, and TN for each study and confirmed to be consistent with the originally reported values in the source publications.

To minimize the risk of double-counting patients across studies originating from overlapping institutions, time periods, or research groups, we performed corresponding verification procedures. For studies sharing any common authors, institutions, or recruitment periods, we cross-referenced sample sizes, enrollment dates, patient demographics (age, sex, and stage distribution), and imaging protocols to assess whether patient cohorts were independent.

### Outcome Measures

The primary outcome measures were the sensitivity, specificity, and AUC of radiomics-based AI models for predicting pCR and MPR to neoadjuvant immunochemotherapy in NSCLC. Secondary outcomes included the predictive performance of radiological response criteria (PERCIST and RECIST 1.1) for pCR and MPR, as well as subgroup analyses across different validation sets (internal vs external), imaging modalities (CT vs PET/CT), and specific AI algorithms. For radiomics-based AI, data were extracted exclusively from internal or external validation cohorts. If a study reported multiple nonoverlapping validation cohorts, their contingency tables were considered independent, and all were extracted. However, for studies presenting multiple AI models derived from the same patient population, only the primary model was included. The primary model was operationally defined using the following hierarchical criteria: (1) the model designated by the original study authors as the primary or final model in their abstract or conclusion; (2) if not explicitly designated, the model with the highest AUC in the primary validation cohort. We acknowledge that this approach may introduce optimism bias, as the model achieving the highest performance in a given dataset does not necessarily represent the most generalizable model.

### Statistical Analysis

#### Assessment of Threshold Effects

To evaluate whether variation in decision thresholds across included AI models contributed to heterogeneity, we log-transformed sensitivity and specificity and examined their correlation using the Spearman rank correlation coefficient. Given the inherent interstudy heterogeneity, a bivariate random-effects model was employed to estimate pooled sensitivity, specificity, and AUC [16]. The threshold for clinical significance is defined by whether the value crosses 0.80 as the cutoff point. Statistical differences between subgroups were evaluated using the *Z* test, with significance defined as *P*<.05. Heterogeneity was quantified using the Higgins and *I*² statistic [17]; for models exhibiting substantial heterogeneity (*I*²>50%), bivariate box plots and multivariate meta-regression were utilized to explore potential sources of variance. Subgroup analyses were visualized through violin plots for validation cohorts and imaging modalities, while subgroup summary receiver operating characteristic curves were utilized to compare algorithmic performance. Radar charts and bubble plots were generated to illustrate algorithm distribution frequencies and temporal development trends, respectively. The clinical utility of the AI models was evaluated using the Fagan nomogram, using the median prevalence of the included studies as the pretest probability. Potential publication bias was assessed using the Deeks funnel plot asymmetry test, with a threshold of *P*<.10 indicating significant bias [18]. Statistical computations were performed using Stata 15.1 (StataCorp LLC; Midas and Metadata packages), MetaDiSc 1.4 (Clinical Biostatistics Unit, Hospital Ramón y Caja), and R version 4.5.1 (R Foundation for Statistical Computing; ggplot2 and tidyverse packages). All subgroup analyses—including stratification by imaging modality (CT vs PET/CT), validation type (internal vs external), and AI architecture (machine learning vs deep learning)—were prespecified in the PROSPERO protocol (CRD420251243962). Subgroup analyses performed using specific algorithm types (eg, support vector machine, convolutional neural network, and least absolute shrinkage and selection operator) were not prespecified and are therefore presented as exploratory.

### Ethical Considerations

As this is a systematic review and meta-analysis, ethics approval and consent to participate are not applicable.

## Results

### Study Selection

**Figure 1.**
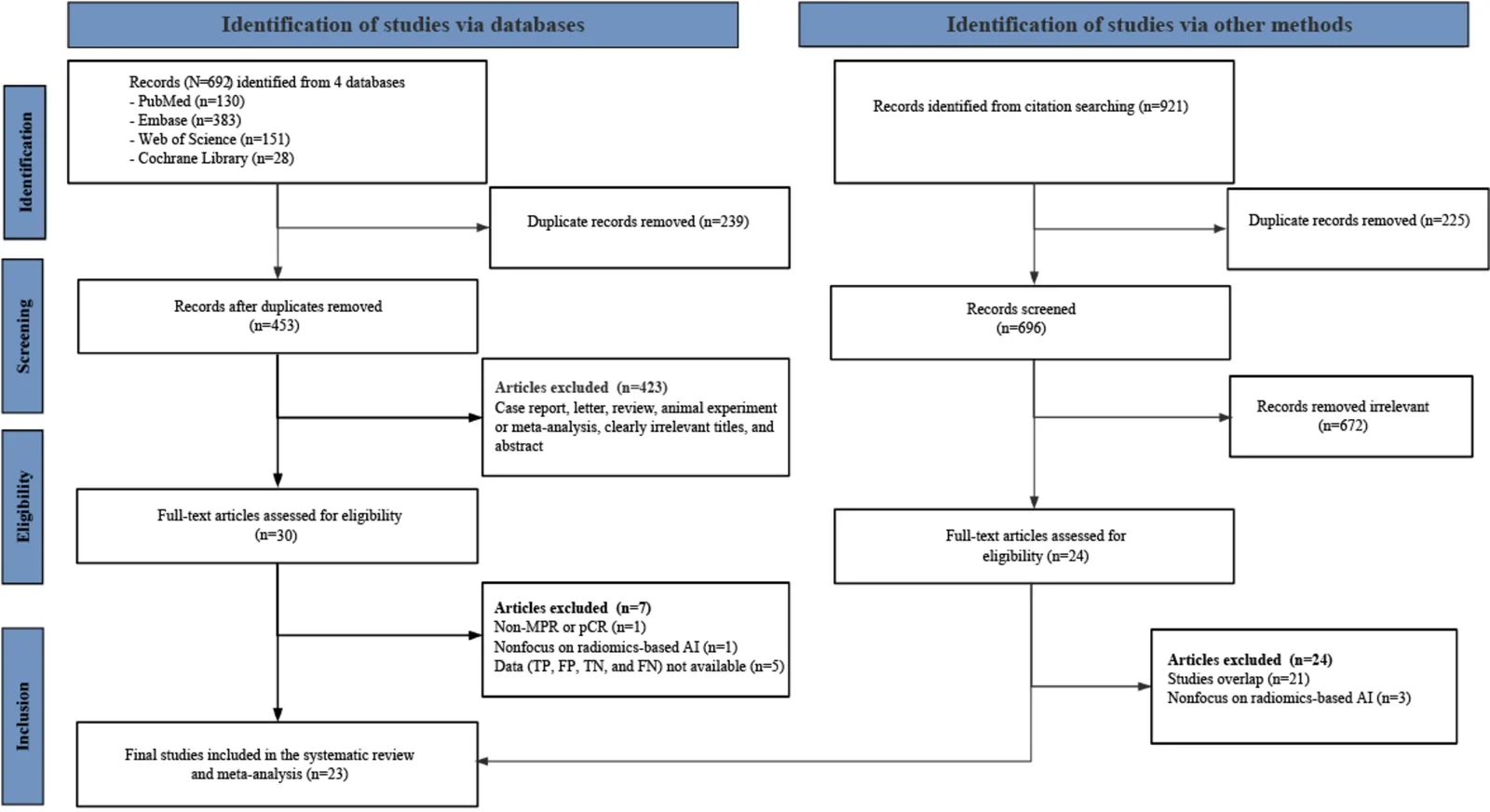
Flow diagram of the study selection process for the systematic review and meta-analysis. FN: false negatives; FP: false positives; MPR: major pathological response; NSCLC: non–small cell lung cancer; pCR: pathological complete response; TN: true negatives; TP: true positives.

The initial systematic search across the designated databases yielded a total of 692 potentially relevant records. Following the removal of 239 duplicate citations, 453 unique records underwent preliminary screening. During this phase, 423 articles were excluded based on title and abstract review due to irrelevance or inappropriate publication types, leaving 30 studies for comprehensive full-text evaluation. Upon detailed assessment, 7 studies were further excluded for the following reasons: 5 lacked sufficient or complete diagnostic data (TP, FP, FN, and TN) required for quantitative synthesis; 1 did not utilize a radiomics-based AI methodology; and 1 failed to employ MPR or pCR as end points. Consequently, 23 studies met all eligibility criteria and were included in the final meta-analysis [5,6,10-12,19-36]. The selection process was conducted in strict accordance with the PRISMA guidelines, as delineated in the flow diagram (Figure 1).

### Study Description

The final synthesis included 13 studies focusing on pCR [12,20-28,30,31,35], encompassing a total of 1031 patients across various validation cohorts. Within this subset, 11 studies utilized internal validation, while 5 incorporated external validation sets. Regarding imaging modalities, 8 studies evaluated CT-based models and 5 utilized PET/CT-based approaches. Logistic regression was the most frequently implemented AI algorithm (Figure S1 in Multimedia Appendix 1). Notably, one study provided a direct comparison between radiomics-based models and radiological response criteria, specifically PERCIST and RECIST 1.1 [35].

For the MPR end point, 10 studies were included [5,6,10,11,19,29,32-34,36], representing 973 patients in validation cohorts. This comprised 9 internal validation and 6 external validation sets. The majority of these investigations (n=8) focused on CT-based imaging, while 2 utilized PET/CT. Convolutional neural networks emerged as the predominant algorithmic approach (Figure S2 in Multimedia Appendix 1). Two studies included comparative performance data for the RECIST 1.1 criteria [33,36]. Comprehensive details regarding patient demographics, raw diagnostic data, and imaging parameters are systematically documented in Table 1 and Tables S5–S9 in see Multimedia Appendix 1.

**Table 1.** Study and patient characteristics of the included studies.

Author and year	Country	Study design	Target condition	Reference standard	Analysis	Total number of patients			Number of response patients (D+)
						Training	Internal validation	External validation	
Deng et al [35] (2025)	China	Retro^a^	pCR^b^	Pathology	PB^c^	36	—^d^	8	pCR: training: 19, external validation: 8
Yang et al [23] (2025)	China	Retro	pCR	Pathology	PB	137	92	—	pCR: training: 57, internal validation: 40
Sheng et al [27] (2025)	China	Retro	pCR	Pathology	PB	144	61	—	pCR: training: 65, internal validation: 26
Liu et al [31] (2024)	China	Retro	pCR	Pathology	PB	74	32	—	pCR: training: 21, internal validation: 14
Liu et al [30] (2025)	China	Retro	pCR	Pathology	PB	147	63	—	pCR: training: 48, internal validation: 21
Yang et al [24] (2023)	China	Retro	pCR	Pathology	PB	77	33	—	pCR: training: 21, internal validation: 18
Qu et al [28] (2024)	China	Retro	pCR	Pathology	PB	104	69	75	pCR: training: 25, internal validation: 24, external validation: 24
Ye et al [21] (2024)	China	Retro	pCR	Pathology	PB	91	22	112	pCR: training: 32, internal validation: 8, external validation: 40
Fan et al [12] (2025)	China	Retro	pCR	Pathology	PB	151	65	—	pCR: training: 78, internal validation: 33
Ye et al [20] (2024)	China	Retro	pCR	Pathology	PB	108	—	70	pCR: training: 41, external validation: 23
Ye et al [22] (2025)	China	Retro	pCR	Pathology	PB	308	78	148	pCR: training: 120, internal validation: 30, external validation: 52
Yang et al [26] (2025)	China	Retro	pCR	Pathology	PB	78	19	—	pCR: training: 26, internal validation: 7
Yang et al [25] (2024)	China	Retro	pCR	Pathology	PB	101	84	—	pCR: training: 26, external validation: 28
Bao et al [36] (2025)	China	Retro	MPR^e^	Pathology	PB	186	80	86	MPR: training: 98, internal validation: 42, external validation: 56
Ma et al [6] (2025)	China	Retro	MPR	Pathology	PB	82	33	—	MPR: training: 52, internal validation: 21
Zheng et al [5] (2025)	China	Retro	MPR	Pathology	PB	357	50	59	MPR: training: 195, internal validation: 26, external validation: 30
Han et al [34] (2025)	China	Retro	MPR	Pathology	PB	146	61	36	MPR: training: 100, internal validation: 43, external validation: 23
Liu et al [32] (2023)	China	Retro	MPR	Pathology	PB	64	25	—	pCR: training: 40, internal validation: 16
Geng et al [10] (2025)	China	Retro	MPR	Pathology	PB	172	44	116	MPR: training: 74, internal validation: 22, external validation: 66
Zhang et al [19] (2025)	China	Retro	MPR	Pathology	PB	212	85	—	MPR: training: 100, external validation: 30
Han et al [33] (2024)	China	Retro	MPR	Pathology	PB	114	50	—	MPR: training: 54, internal validation: 26
Peng et al [29] (2025)	China	Retro	MPR	Pathology	PB	200	NA	60	MPR: training: 96, external validation: 32
Gan et al [11] (2025)	China	Retro	MPR	Pathology	PB	83	35	153	MPR: training: 38, internal validation: 18, external validation: 94

a Retro: retrospective.

b pCR: pathological complete response.

c PB: patient-based.

d Not available.

e MPR: major pathological response.

### Quality Assessment and GRADE Certainty

**Figure 2.**
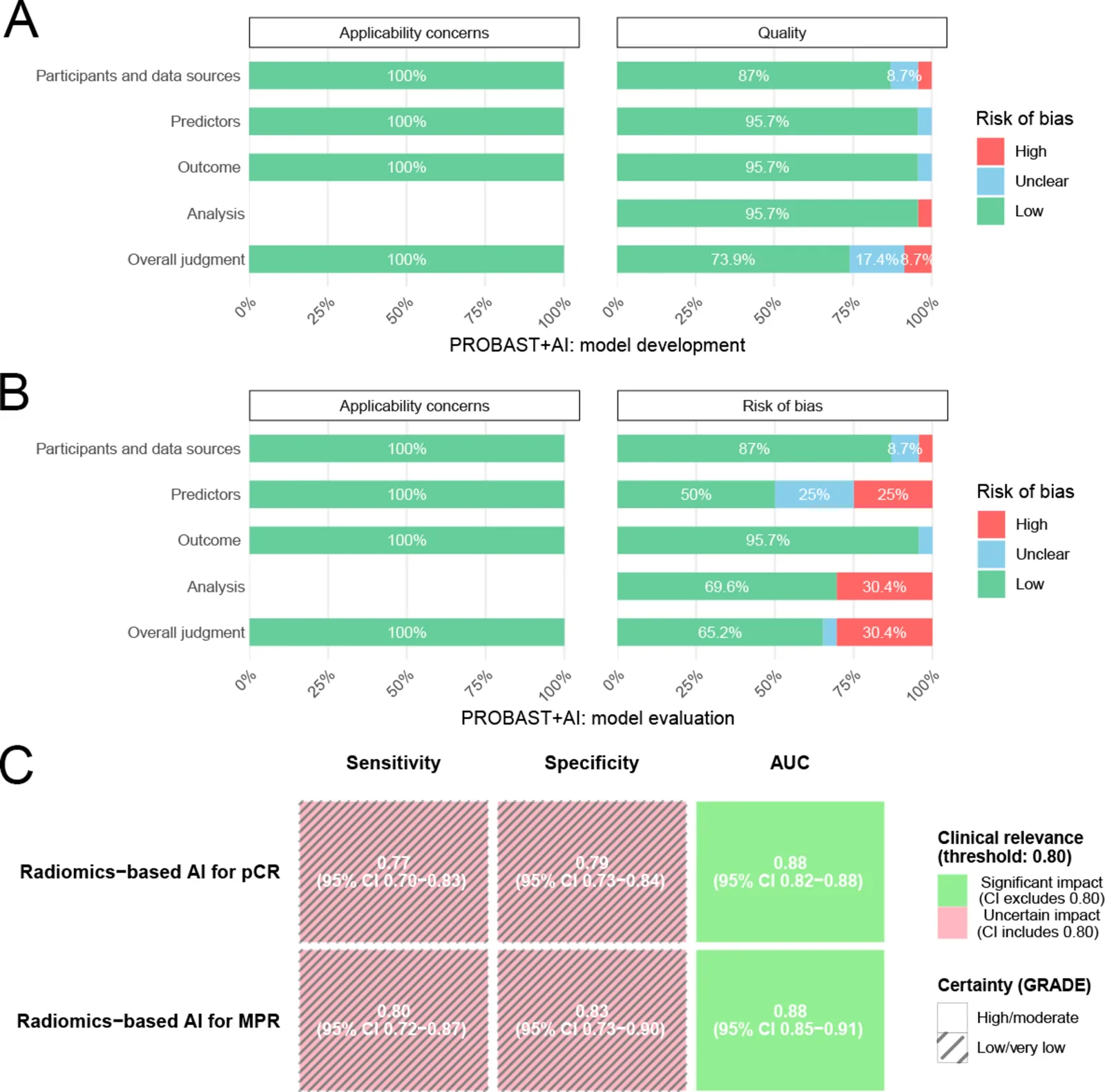
Risk of bias assessment and certainty of evidence. (A) Summary of risk of bias and applicability concerns for model development using PROBAST (Prediction model Risk of Bias Assessment Tool)+AI. (B) Summary of risk of bias and applicability concerns for model evaluation. (C) Summary of findings for predicting pathological complete response (pCR) and major pathological response (MPR), presenting pooled sensitivity, specificity, and area under the curve (AUC), with clinical relevance and certainty of evidence ratings. GRADE: Grading of Recommendations Assessment, Development and Evaluation.

The methodological quality of the included studies, assessed via the PROBAST+AI tool, is summarized in Figure 2A and B and Tables S2 and S3 in Multimedia Appendix 1. Regarding the model development phase, 8% (2/23) of the studies were classified as having a high risk of bias, while no studies raised high concerns regarding applicability. For the model validation phase, the overall risk of bias was rated as high in 30% (7/23) of the studies, though concerns regarding applicability remained low across the cohort. Collectively, the prevalence of high-risk domains was limited, with the majority of studies exhibiting a low risk of bias, indicating that the overall methodological quality of the included literature is acceptable for meta-analytical synthesis. The certainty of evidence for the diagnostic outcomes was evaluated using the GRADE framework, with the quality of evidence ranging from low to moderate (Table S4 in Multimedia Appendix 1). This suggests a relatively weak level of certainty in the pooled estimates. The primary factors contributing to the downgrading of evidence were risks of bias.

To gage the transparency and reproducibility of the included AI studies, we systematically assessed 5 key reporting dimensions: calibration, external validation, explainability methods, availability of code or trained models, and reference to established AI reporting guidelines (TRIPOD-AI [Transparent Information for Potential Outcomes and Diagnostics-AI] or CONSORT-AI [Consolidated Standards of Reporting Trials-AI]). Fourteen of 23 (61%) studies reported calibration assessment, predominantly via calibration plots, with 4 studies additionally performing the Hosmer-Lemeshow test. Eleven (48%) studies employed external validation. Specific explainability methods—such as SHAP (Shapley Additive Explanations) values (n=19), Grad-CAM (gradient-weighted class activation mapping) heatmaps (n=4), attention maps (n=3), saliency maps (n=2), and feature importance analysis (n=2)—were reported in 19 (83%) studies. Three (13%) studies made code or trained model weights publicly available via GitHub repositories, and 6 (26%) studies explicitly referenced TRIPOD-AI or CONSORT-AI reporting guidelines. These findings are summarized in Table S10 in Multimedia Appendix 1.

### Radiomics-Based AI vs Radiological Response Criteria

**Figure 3.**
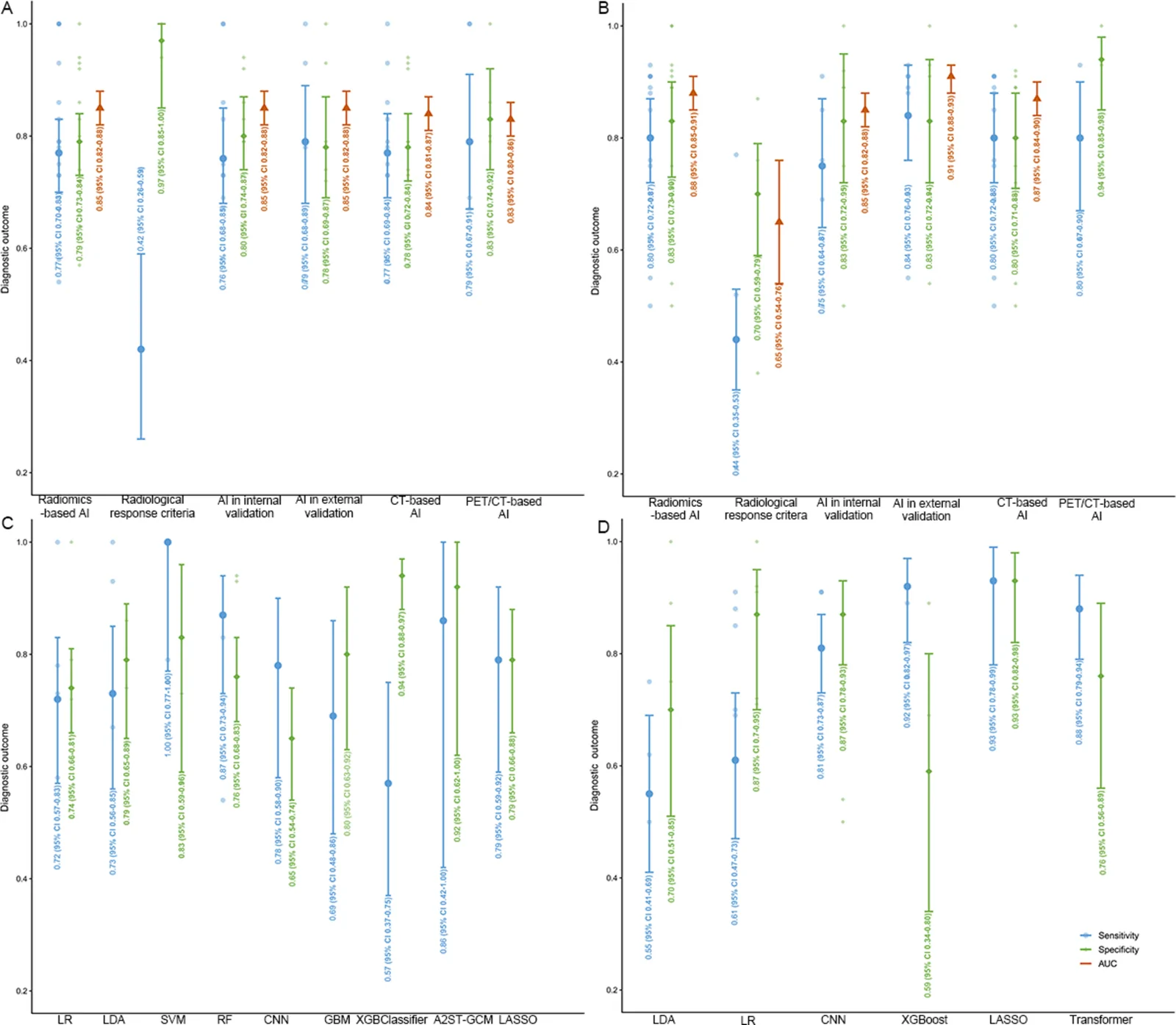
Violin plots of the diagnostic performance for pathological response. The upper panels show the overall distribution of diagnostic outcomes for (A) pathological complete response (pCR) and (B) major pathological response (MPR) across all included studies. The lower panels compare the distribution of diagnostic outcomes based on different AI algorithms for (C) pCR and (D) MPR. A2ST-GCM: adaptive attention-guided spatiotemporal graph convolutional network; AUC: area under the curve; CNN: convolutional neural network; CT: computed tomography; GBM: gradient boosting machine; LASSO: least absolute shrinkage and selection operator; LDA: linear discriminant analysis; LR: logistic regression; PET/CT: positron emission tomography/computed tomography; RF: random forest; SVM: support vector machine; XGBoost: extreme gradient boosting.

The Spearman correlation coefficient between logit-transformed sensitivity and specificity was 0.09 (*P*=.77) for pCR and −0.12 (*P*=.72) for MPR. In the prediction of pCR, the radiomics-based AI models demonstrated a pooled sensitivity of 0.77 (95% CI 0.70‐0.83, *I*^2^=59%; low certainty) and a specificity of 0.79 (95% CI 0.73‐0.84, *I*^2^=67%; low certainty; Figure S3 in Multimedia Appendix 1), with a summary AUC of 0.85 (95% CI 0.82‐0.88; moderate certainty; Figure S4 in Multimedia Appendix 1), as illustrated in Figures 2C and 3A. Notably, the sensitivity of the AI-based approach was significantly superior to that of radiological response criteria (0.77 vs 0.42; *Z*=3.97, *P*<.001), according to Table S11 and Figures S5 and S6 in Multimedia Appendix 1. After excluding the 2 potentially overlapping studies by Ye et al [20, 21], the summary receiver operating characteristic analysis for predicting pCR in the remaining studies yielded a sensitivity of 0.73 (95% CI 0.66‐0.80) and a specificity of 0.82 (95% CI 0.75‐0.87), with an AUC of 0.83 (95% CI 0.80‐0.86). These estimates showed no substantial change from those prior to exclusion, as illustrated in Figure S7 in Multimedia Appendix 1.

Regarding MPR prediction, radiomics-based AI yielded a pooled sensitivity of 0.80 (95% CI 0.72‐0.87, *I*^2^=79%; low certainty) and a specificity of 0.83 (95% CI 0.73‐0.90, *I*^2^=73%; low certainty; Figure S8 in Multimedia Appendix 1), with an AUC of 0.88 (95% CI 0.85‐0.91; moderate certainty; Figure S9 in Multimedia Appendix 1), as shown in Figures 2C and 3B. The sensitivity and AUC of the radiomics-based AI were significantly higher than those of the traditional clinical model (0.80 vs 0.44; *Z*=6.02, *P*<.001 and 0.88 vs 0.65; *Z*=3.95, *P*<.001, respectively), as shown in Table S12 and Figures S10-S12 in Multimedia Appendix 1.

### Subgroup Analysis for Different Algorithms

**Figure 4.**
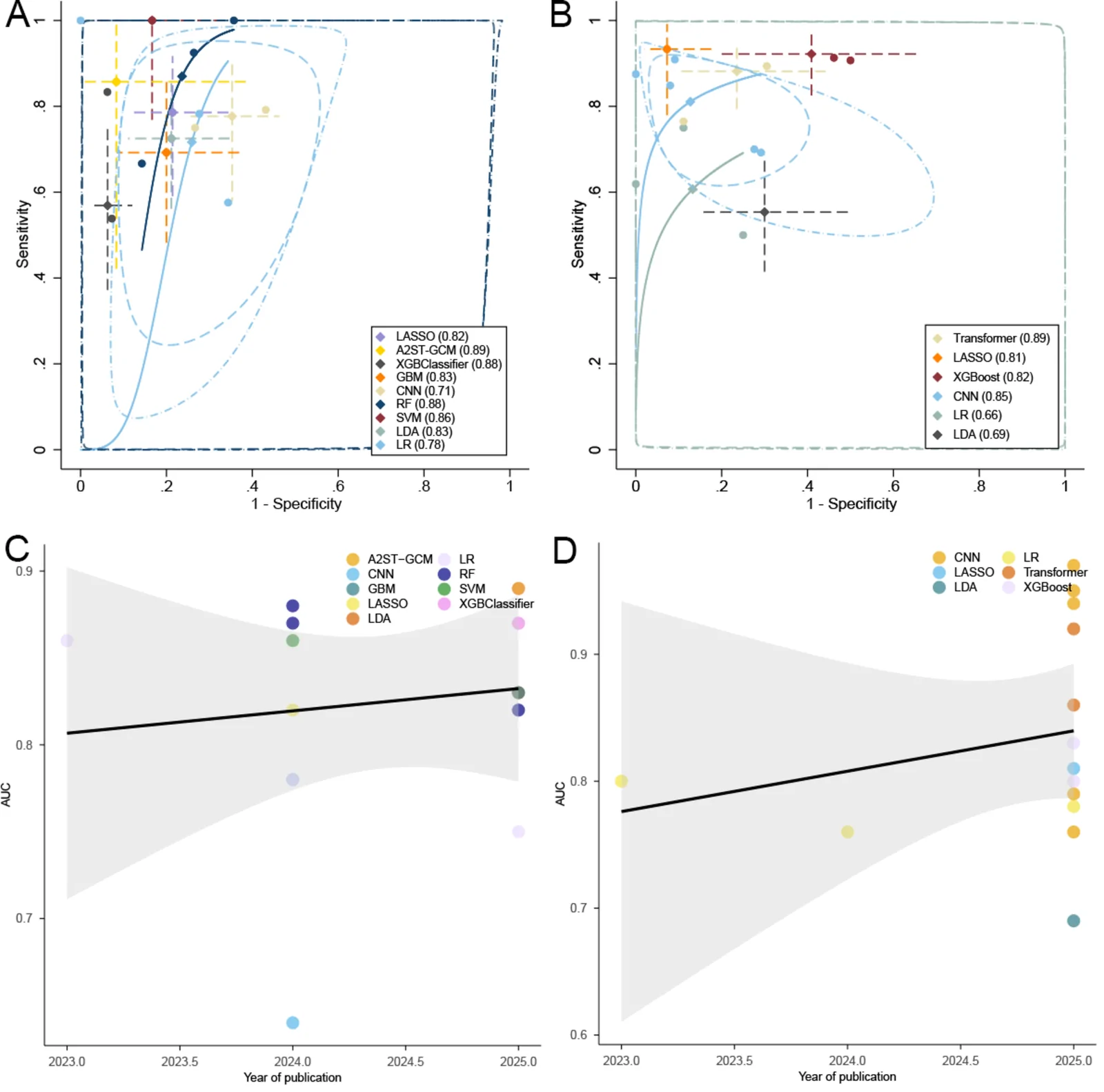
Comprehensive diagnostic performance plots. The upper panels display the subgroup summary receiver operating characteristic (SROC) curves comparing different AI algorithms for (A) pathological complete response (pCR) and (B) major pathological response (MPR). The lower panels display the bubble plots comparing different AI algorithms and showing the relationship between study characteristics (typically represented by bubble size or color) and diagnostic performance for (C) pCR and (D) MPR. AUC: area under the curve; A2ST-GCM: adaptive attention-guided spatiotemporal graph convolutional network; CNN: convolutional neural network; GBM: gradient boosting machine; LASSO: least absolute shrinkage and selection operator; LDA: linear discriminant analysis; LR: logistic regression; RF: random forest; SVM: support vector machine; XGBoost: extreme gradient boosting.

For pCR prediction (Figures 3C and 4A), support vector machine achieved the highest pooled sensitivity (1.00, 95% CI 0.77‐1.00), and XGBClassifier achieved the highest specificity (0.94, 95% CI 0.88‐0.97), while A2ST-GCM (adaptive attention-guided spatiotemporal graph convolutional network) yielded the top AUC of 0.89, according to Figure S13 in Multimedia Appendix 1. For MPR (Figures 3D and 4B), least absolute shrinkage and selection operator led to both sensitivity (0.93, 95% CI 0.78‐0.99) and specificity (0.93, 95% CI 0.82‐0.98), with the transformer model achieving the highest AUC (0.89), according to Figure S14 in Multimedia Appendix 1. Bubble plots (Figure 4C-D) indicated a temporal trend of improving accuracy for both outcomes.

### Subgroup Analysis for Different Validations and Image Types

For pCR, the meta-regression analysis included 10 internal validation datasets (n=618) and 6 external validation datasets (n=413). Pooled sensitivity was 0.76 (95% CI 0.68‐0.85) for internal and 0.79 (95% CI 0.68‐0.89) for external validation (*Z*=0.44, *P*=.66); pooled specificity was 0.80 (95% CI 0.74‐0.87) for internal and 0.78 (95% CI 0.69‐0.87) for external validation (*Z*=0.35, *P*=.72), and AUC was 0.85 (95% CI 0.82‐0.88) for both (*Z*=0.00, *P*>.99), according to Figures S15 and S16 in Multimedia Appendix 1. Similarly, comparisons between CT-based (n=11) and PET/CT-based (n=5) models showed no significant differences (all *P*>.05), indicating robust diagnostic performance across validation types and imaging modalities for pCR prediction, according to Figures S17 and S18 in Multimedia Appendix 1. For MPR, 7 internal (n=463) and 7 external (n=510) validation datasets were compared. Pooled sensitivity was 0.75 (95% CI 0.64‐0.87) for internal and 0.84 (95% CI 0.76‐0.93) for external validation (*Z*=1.22, *P*=.23); pooled specificity was 0.83 (95% CI 0.72‐0.95) for internal and 0.83 (95% CI 0.72‐0.94) for external validation (*Z*=0.00, *P*>.99), and AUC was significantly higher in external validation cohorts (0.91, 95% CI 0.88‐0.93) compared with internal sets (0.85, 95% CI 0.82‐0.88; *Z*=3.01, *P*=.002), according to Figures S19 and S20 in Multimedia Appendix 1. Additionally, PET/CT models demonstrated superior specificity compared to CT (0.95 vs 0.80; *Z*=2.82, *P*=.005). However, this finding was based on only 2 PET/CT studies and should therefore be interpreted as hypothesis-generating rather than confirmatory. These results are presented in Figure 3 and Tables S14 and S15 and Figures S21–S23 in Multimedia Appendix 1).

### Heterogeneity Analysis: Bivariate Box Plots and Meta-Regression

**Figure 5.**
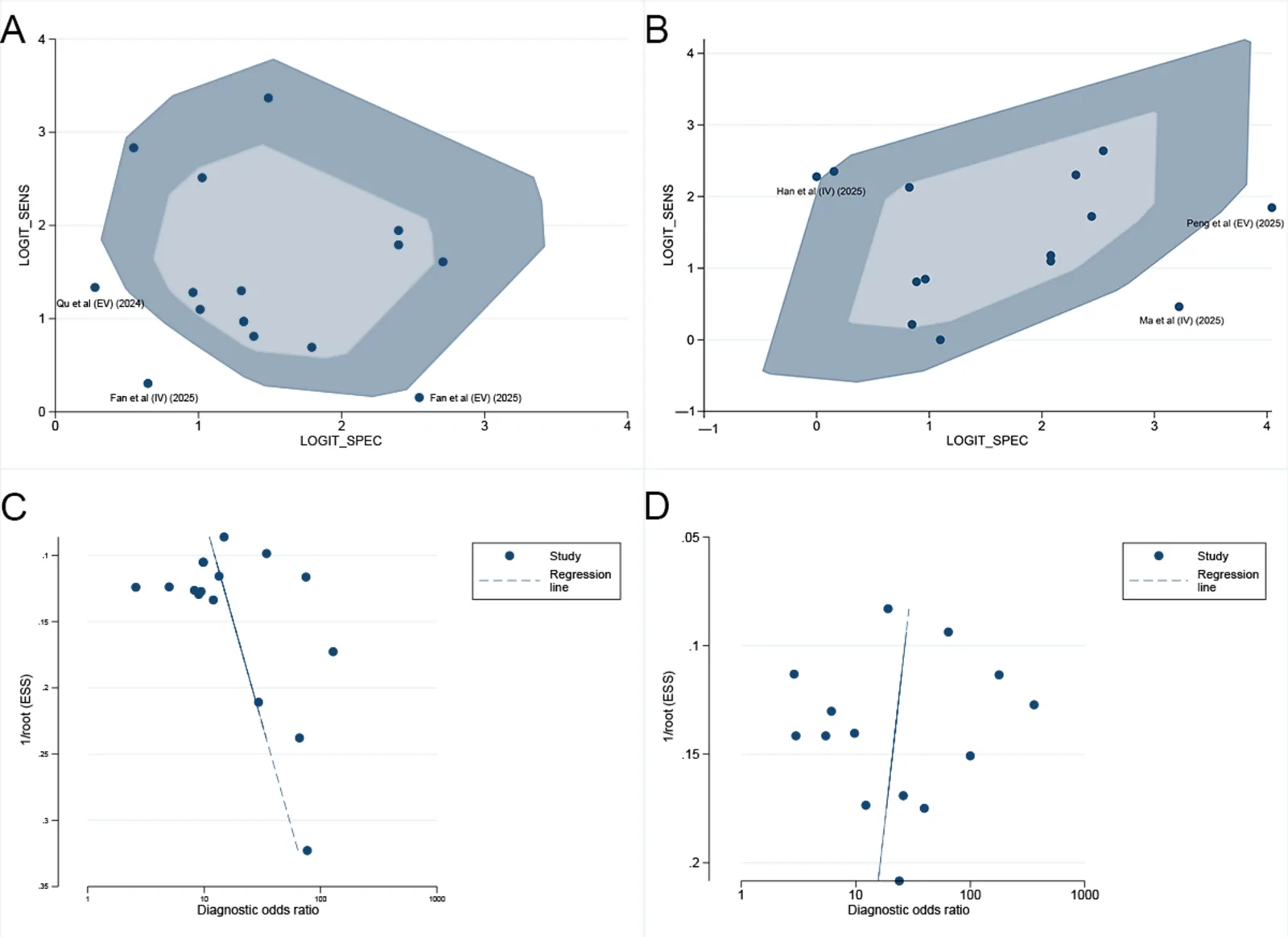
Bivariate box plots and funnel plots of the diagnostic performance for pathological response. The upper panels display the bivariate box plots of sensitivity and specificity for (A) pathological complete response (pCR) and (B) major pathological response (MPR). The lower panels display the Deeks funnel plots for assessing publication bias for (C) pCR, *P*=.30 and (D) MPR, *P*=.72. ESS: effective sample size; EV: external validation; IV: internal validation.

Bivariate box plots and univariate meta-regression analyses were conducted to investigate potential sources of heterogeneity in the diagnostic performance estimates. For the prediction of pCR, meta-regression suggested that the type of AI model (radiomics model vs radiomics and clinical model) might be a source of heterogeneity (Table S13 in Multimedia Appendix 1). The corresponding bivariate box plot indicated that the studies by Fan et al [12] and Qu et al [28] were potential outliers, possibly contributing to heterogeneity among AI algorithms (Figure 5A). Regarding MPR prediction, meta-regression identified the AI method (machine learning vs deep learning) as a potential contributor to heterogeneity (Table S14 in Multimedia Appendix 1). The bivariate box plot for this outcome highlighted the studies by Ma et al [6], Peng et al [29], and Han et al [34] as potential outliers, particularly within the deep learning subgroup (Figure 5B).

### Publication Bias and Clinical Application Value

**Figure 6.**
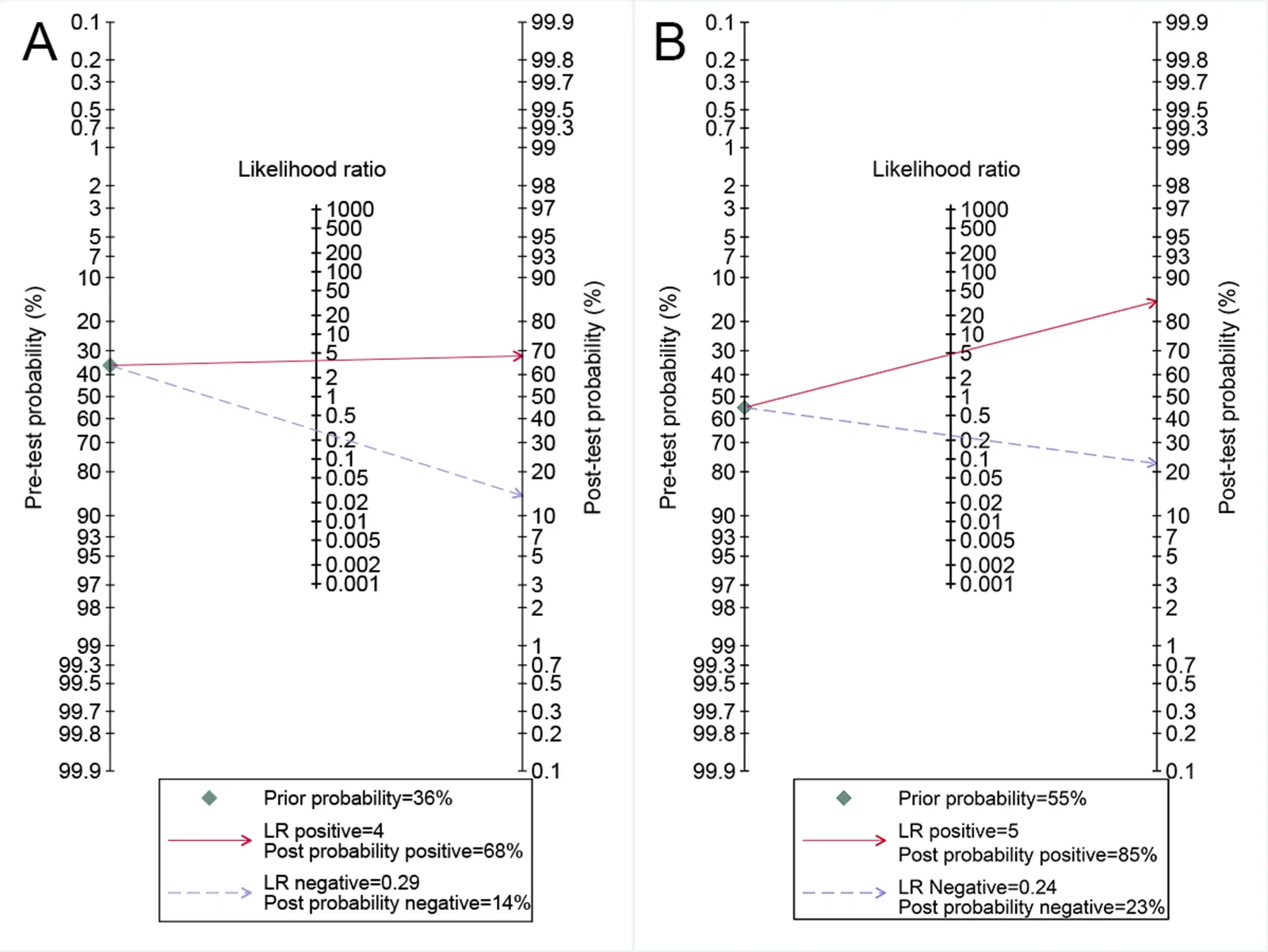
Fagan plots for assessing the clinical utility of radiomics-based AI. The plots show the posttest probability of pathological response based on pretest probability and the likelihood ratio for (A) pathological complete response (pCR) and (B) major pathological response (MPR). LR: logistic regression.

The Deeks funnel plot asymmetry test showed no significant publication bias for either pCR (*P*=.30) or MPR (*P*=.72; Figure 5C–D). Using a median disease incidence, Fagan nomograms demonstrated clinical utility: for pCR (36% prior probability), the positive and negative posttest probabilities were 68% and 14%, respectively (Figure 6A). Similarly, for MPR (55% prior probability), these values were 85% and 23%, respectively (Figure 6B).

## Discussion

### Principal Findings

The primary finding of this meta-analysis indicates that radiomics-based AI significantly outperforms traditional radiological response criteria (eg, RECIST 1.1 and PERCIST) in predicting both pCR and MPR following neoadjuvant immunochemotherapy for NSCLC. Specifically, AI models demonstrated superior sensitivity for pCR, as well as both sensitivity and AUC for MPR. These results align with emerging evidence across solid tumors, suggesting that traditional size-based metrics systematically underestimate the true pathological benefit of immunotherapy [37]. Mechanistically, the superiority of AI likely stems from its ability to decode subvisual tumor heterogeneity. While RECIST relies on linear diameter reduction—often misclassifying volume-stable but biologically inactivated lesions (eg, scar tissue and necrosis) as “stable disease,” AI models extract high-dimensional quantitative features from raw images [38,39]. These features, including texture, wavelet transformations, and delta-radiomics signatures, capture microstructural alterations such as treatment-induced necrosis, fibrosis, and immune infiltration [40,41].

Subgroup analysis stratified by imaging modality highlighted a critical distinction: PET/CT-based models demonstrated significantly superior specificity compared to CT-based models for identifying MPR. This finding highlights the distinct advantage of integrating functional metabolic data with anatomical information to effectively exclude patients who do not achieve MPR. In contrast, CT-based radiomics may misclassify posttreatment fibrosis or inflammation as residual tumor, leading to FPs, whereas ^18^F-FDG (fluorine-18 fluorodeoxyglucose) PET directly characterizes tumor cell viability and metabolic heterogeneity [42,43]. Thus, PET/CT models may offer greater reliability in excluding nonresponders, providing robust evidence to guide decisions regarding surgical timing or organ preservation strategies.

Regarding algorithmic performance, advanced deep learning architectures showed distinct advantages. The graph convolutional network (eg, A2ST-GCM) and transformer-based models achieved the highest predictive performance for pCR and MPR, respectively. The success of graph convolutional networks likely lies in their ability to model nonlinear anatomical dynamics through adaptive spatiotemporal topology, surpassing rigid pixel-wise correspondence [23]. Conversely, transformer architectures excel in multimodal fusion and capturing global long-range dependencies, facilitating the integration of macroscopic radiomics with microscopic features to discern the nuanced threshold of residual viable tumor [11].

### Implications for Practice and Research

To illustrate the clinical relevance of the observed diagnostic divergence between AI and conventional criteria, consider 2 prototypical scenarios. In the first scenario, a patient with stage IIIA NSCLC exhibits a 25% reduction in tumor diameter by RECIST 1.1 after neoadjuvant immunochemotherapy, meeting the threshold for partial response. However, AI-based analysis of the same CT reveals extensive treatment-induced fibrosis with sparse residual tumor nests—features beyond human visual detection—and predicts a high probability of MPR. In this discordant case, reliance on RECIST alone would underestimate the depth of pathological response, potentially delaying surgery or prompting unnecessary additional therapy. AI-informed assessment could instead support timely surgical resection. In the second scenario, a patient demonstrates stable disease by RECIST, but AI predicts a low probability of pathological response. Here, the discordance may reflect true residual viable tumor that anatomical criteria fail to capture as progression, and the AI prediction could prompt early reevaluation, repeat biopsy, or consideration of alternative therapeutic strategies. These scenarios underscore that AI and conventional criteria provide complementary rather than redundant information, and their combined use—rather than the replacement of one by the other—may optimize clinical decision-making. However, as only a limited number of included studies directly compared AI against RECIST/PERCIST, these scenarios remain illustrative; future head-to-head comparative studies are needed to quantify the frequency and clinical impact of such discordance.

From a patient communication perspective, Fagan nomograms offer a clinically intuitive tool for translating AI-derived likelihood ratios into individualized posttest probabilities of pathological response. For example, given a pretest probability of 36% for pCR (the median prevalence across included studies), a positive AI prediction raises the posttest probability to 68%, while a negative prediction reduces it to 14%. These probabilistic outputs can be directly incorporated into shared decision-making conversations: a patient with a 68% posttest probability of pCR may be more informed when considering surgical timing or organ-preservation strategies, whereas a 14% posttest probability may prompt the discussion of alternative neoadjuvant regimens. Future work should evaluate the impact of AI-assisted risk communication on decisional quality, patient anxiety, and treatment adherence in the neoadjuvant setting.

There are several strengths to this study. To our knowledge, this is the first systematic review and meta-analysis to evaluate radiomics-based AI for predicting pCR and MPR in patients with NSCLC undergoing neoadjuvant immunochemotherapy. A distinguishing feature of this work is the direct comparison of AI models against standard radiological criteria (RECIST 1.1 and PERCIST). By juxtaposing these diagnostic metrics, we provide current evidence of the clinical potential of radiomics-based AI. We prioritized methodological rigor to maximize translational relevance; specifically, we adhered to the recently updated PROBAST+ AI framework to systematically appraise bias and applicability, complemented by the GRADE approach to transparently rate the certainty of evidence. Beyond global performance metrics, we conducted comprehensive subgroup analyses stratified by imaging modality, validation type, and algorithmic architecture. This granular evaluation enabled us to dissect sources of heterogeneity and identify key factors driving diagnostic accuracy.

To address the substantial heterogeneity often observed in AI meta-analyses, we used a bivariate random-effects model coupled with meta-regression. The results indicated that, for pCR prediction, differences in AI models (radiomics model vs radiomics and clinical model) represent a potential source of heterogeneity. While pure radiomics models excel at characterizing structural phenotypes and the immune microenvironment, the integration of clinical variables—such as stage, histology, and inflammatory indices—provides significant complementary values [20,44,45]. For MPR prediction, the distinction between AI architectures (machine learning vs deep learning) emerged as a key factor. Although traditional machine learning offers stability via handcrafted features, deep learning captures more complex spatial patterns to achieve superior performance, which in turn contributes to greater between-study variance [21,46].

Beyond diagnostic accuracy metrics, the translation of radiomics-based AI into clinical practice requires alignment with digital health infrastructure. These models are designed to operate as embedded components within digital oncology workflows—for example, as automated preprocessing modules in preoperative decision support systems that generate real-time probabilistic predictions of pathological response, as standardized inputs for multidisciplinary virtual tumor board discussions, and as sharable risk assessments within teleconsultation platforms connecting tertiary centers with community hospitals [19]. Such integration demands not only robust model performance but also interoperability with clinical information systems, adherence to data governance frameworks, and compliance with regulatory standards for software as a medical device [47,48]. The present findings, by establishing benchmark diagnostic performance, constitute a foundational step toward the development and regulatory evaluation of these digital health tools.

### Heterogeneity

The pooled estimates for both pCR and MPR exhibited substantial between-study heterogeneity (*I*² ranging from 59% to 79%), and GRADE certainty ratings were downgraded primarily due to the risk of bias arising from the retrospective nature of the primary studies. These findings carry direct clinical implications: at present, radiomics-based AI models should be positioned as supportive decision aids rather than stand-alone diagnostic arbiters. In clinical contexts where a high negative predictive value is prioritized, such as screening patients for organ-preservation strategies or identifying candidates who may safely proceed to surgery without delay, the current sensitivity of approximately 0.80 may already provide a meaningful clinical value, particularly when integrated with multidisciplinary assessment. However, in scenarios where FPs carry severe consequences, such as decisions to forgo surgery based on a predicted complete response, the moderate specificity (0.79‐0.83) and low certainty of evidence argue against sole reliance on AI predictions. The gap between promising research performance and sufficient clinical reliability remains substantial, and bridging it requires prospective validation, standardized imaging protocols, and transparent reporting of model limitations. Furthermore, the absence of calibration reporting and decision-curve analysis in the majority of included studies represents a major limitation in translating discrimination metrics (AUC, sensitivity, and specificity) into clinical decision support, as well-calibrated probability estimates are a prerequisite for tools such as Fagan nomograms to generate clinically actionable posttest probabilities. Final treatment selection remains the purview of clinicians based on specific patient conditions, with AI serving as an embedded component within the decision-making workflow. The clinical implementation faces constraints, including the scarcity of expert-annotated data, regulatory hurdles, and technical barriers regarding data availability, interpretability, and transparency. Progress in few-shot learning, self-supervised models, and centralized platforms, coupled with more transparent algorithms, offers pathways to integrate the AI ecosystem, thereby providing comprehensive solutions that genuinely augment clinical practice [49,50].

### Limitations

Several limitations of the current meta-analysis should be considered when interpreting the results, which we categorize into three domains. (1) Study-level limitations: retrospective designs. All included studies employed a retrospective design, which inherently introduces potential selection and information biases. The absence of prospective validation cohorts limits the generalizability of the pooled estimates to real-world clinical settings, where patient populations, imaging protocols, and pathological assessment procedures are more heterogeneous. (2) Model-level limitations: best-performing model selection and calibration gaps. To avoid statistical redundancy associated with patient overlap, data extraction was restricted to the single AI algorithm demonstrating the highest performance within each study. This approach was operationalized by selecting the model with the highest reported AUC in the primary validation cohort. This methodological choice entails a risk of optimism bias, as the best-performing model on a given dataset may not represent the most generalizable model. (3) System-level limitations: data availability, regulation, and interoperability. At the system level, several barriers limit clinical translation. First, most of the included studies did not provide available code, model weights, or standardized feature extraction pipelines across the included studies, which preclude independent external validation and reproducibility assessment. Second, the integration of these AI models into existing clinical information systems and radiology workflows requires standardized data formats (eg, DICOM [Digital Imaging and Communications in Medicine] interoperability) and real-time inference capabilities that were not evaluated in any included study. Based on the evidence gaps identified, we propose the following research priorities: (1) prospective, multicenter validation studies with preregistered protocols and standardized imaging acquisition; (2) head-to-head comparisons of multiple AI models within the same patient cohort to quantify model selection bias; (3) mandatory reporting of calibration, explainability, and code availability in alignment with TRIPOD-AI and CONSORT-AI; and (4) the evaluation of AI model performance within real-world digital health workflows, including the assessment of human-AI interaction, decision impact, and patient outcomes.

### Conclusion

Current evidence indicates that radiomics-based AI demonstrates high diagnostic accuracy and superior sensitivity compared to traditional radiological criteria, showing significant potential for preoperative response assessment. However, the heterogeneity and retrospective design of current studies limit the evidence. Future large-scale, prospective multicenter trials and multimodal data integration are required to validate these findings for clinical translation.

## Supplementary material

10.2196/93892 Multimedia Appendix 1 The detailed search strategy; risk of bias assessments (Prediction Model Risk of Bias Assessment Tool+AI); Grading of Recommendations Assessment, Development and Evaluation scoring; technical aspects and diagnostic raw data; and summary of receiver operating characteristic curves for radiomics-based AI models.

10.2196/93892 Checklist 1 PRISMA-DTA checklist.

## Abbreviations

^18^F-FDG: fluorine-18 fluorodeoxyglucose
A2ST-GCM: adaptive attention-guided spatiotemporal graph convolutional network
AUC: area under the curve
CONSORT-AI: Consolidated Standards of Reporting Trials-AI
CT: computed tomography
DICOM: Digital Imaging and Communications in Medicine
FN: false negative
FP: false positive
Grad-CAM: gradient-weighted class activation mapping
GRADE: Grading of Recommendations Assessment, Development and Evaluation
MPR: major pathological response
NSCLC: non–small cell lung cancer
pCR: pathological complete response
PERCIST: Positron Emission Tomography Response Criteria in Solid Tumors
PET: positron emission tomography
PRISMA-DTA: Preferred Reporting Items for Systematic Reviews and Meta-Analyses for Diagnostic Test Accuracy
PROBAST: Prediction Model Risk of Bias Assessment Tool
PROSPERO: International Prospective Register of Systematic Reviews
RECIST: Response Evaluation Criteria in Solid Tumors
TN: true negative
TP: true positive
TRIPOD: Transparent Information for Potential Outcomes and Diagnostics
