# Supplementary material for: Radiomics-Based AI for Predicting Neoadjuvant Immunochemotherapy Pathological Response in Non–Small Cell Lung Cancer: Systematic Review and Meta-Analysis
Source: J Med Internet Res. 2026 Aug 13;28:e93892. doi: 10.2196/93892 (PMC13472773; doi:10.2196/93892)
Supplement: Supplementary file 1 [file jmir-v28-e93892-s001.docx]

**Table of Contents**

[**Supplementary Table 1.** Search strategy in PubMed, Embase, Cochrane and Web of Science.	4](#_Toc235031296)

[**Supplementary Table 2.** Risk of bias assessment (PROBAST+AI) model development based on seven domains.	6](#_Toc235031297)

[**Supplementary Table 3.** Risk of bias assessment (PROBAST+AI) model evaluation based on seven domains.	9](#_Toc235031298)

[**Supplementary Table 4**. Grading of recommendations, assessment, development, and evaluations (GRADE) scoring assessments in all the pooled outcomes.	12](#_Toc235031299)

[**Supplementary Table 5.** Technical aspects information and diagnostic raw data of included studies for pCR prediction.	13](#_Toc235031300)

[**Supplementary Table 6.** Technical aspects information and diagnostic raw data of included studies for MPR prediction.	14](#_Toc235031301)

[**Supplementary Table 7.** Diagnostic data for different models.	15](#_Toc235031302)

[**Supplementary Table 8.** Technical aspects of included studies for CT.	16](#_Toc235031303)

[**Supplementary Table 9.** Technical aspects of included studies for PET/CT.	17](#_Toc235031304)

[**Supplementary Table 10.** AI reporting transparency assessment across included studies	18](#_Toc235031305)

[**Supplementary Table 11**. Subgroup analysis for performance comparison for pCR.	19](#_Toc235031306)

[**Supplementary Table 12.** Subgroup analysis for performance comparison for MPR.	20](#_Toc235031307)

[**Supplementary Table 13.** Subgroup analysis and meta-regression analysis for pCR**.**	21](#_Toc235031308)

[**Supplementary Table 14.** Subgroup analysis and meta-regression analysis for MPR**.**	22](#_Toc235031309)

[**Supplementary Figure 1.** Radar plot of the relative diagnostic performance of different radiomics-based artificial intelligence algorithms for predicting pathological complete response.	23](#_Toc235031310)

[**Supplementary Figure 2.** Radar plot of the relative diagnostic performance of different radiomics-based artificial intelligence algorithms for predicting major pathological response.	24](#_Toc235031311)

[**Supplementary Figure 3.** Forest plots of the pooled sensitivity and specificity for predicting pathological complete response based on radiomics-based artificial intelligence.	25](#_Toc235031312)

[**Supplementary Figure 4.** Summary receiver operating characteristic curve for the diagnostic performance of radiomics-based artificial intelligence in predicting pathological complete response.	26](#_Toc235031313)

[**Supplementary Figure 5.** Forest plot of the pooled sensitivity for predicting pathological complete response stratified by RECIST 1.1 and PERCIST models.	27](#_Toc235031314)

[**Supplementary Figure 6.** Forest plot of the pooled specificity for predicting pathological complete response stratified by RECIST 1.1 and PERCIST models.	28](#_Toc235031315)

[**Supplementary Figure 7.** Summary receiver operating characteristic curve for the diagnostic performance of radiomics-based artificial intelligence in predicting pCR after the exclusion of studies with potential patient cohort overlap.	29](#_Toc235031316)

[**Supplementary Figure 8.** Forest plots of the pooled sensitivity and specificity for predicting major pathological response based on radiomics-based artificial intelligence.	30](#_Toc235031317)

[**Supplementary Figure 9.** Summary receiver operating characteristic curve for the diagnostic performance of radiomics-based artificial intelligence in predicting major pathological response.	31](#_Toc235031318)

[**Supplementary Figure 10.** Forest plot of the pooled sensitivity for predicting major pathological response stratified by RECIST 1.1 models.	32](#_Toc235031319)

[**Supplementary Figure 11.** Forest plot of the pooled specificity for predicting major pathological response stratified by RECIST 1.1 model.	33](#_Toc235031320)

[**Supplementary Figure 12.** Summary receiver operating characteristic curve for the diagnostic performance of RECIST 1.1 model in predicting major pathological response.	34](#_Toc235031321)

[**Supplementary Figure 13.** Forest plots of the subgroup analysis for sensitivity and specificity of different artificial intelligence algorithms in predicting pathological complete response.	35](#_Toc235031322)

[**Supplementary Figure 14.** Forest plots of the subgroup analysis for sensitivity and specificity of different artificial intelligence algorithms in predicting major pathological response.	36](#_Toc235031323)

[**Supplementary Figure 15.** Summary receiver operating characteristic curve for the diagnostic performance of radiomics-based ai models on internal validation sets in predicting pathological complete response.	37](#_Toc235031324)

[**Supplementary Figure 16.** Summary receiver operating characteristic curve for the diagnostic performance of radiomics-based ai models on external validation sets in predicting pathological complete response.	38](#_Toc235031325)

[**Supplementary Figure 17.** Summary receiver operating characteristic curve of computed tomography-based radiomics models for predicting pathological complete response.	39](#_Toc235031326)

[**Supplementary Figure 18.** Summary receiver operating characteristic curve of positron emission tomography computed tomography-based radiomics models for predicting pathological complete response.	40](#_Toc235031327)

[**Supplementary Figure 19.** Summary receiver operating characteristic curve for the diagnostic performance of radiomics-based ai models on internal validation sets in predicting major pathological response.	41](#_Toc235031328)

[**Supplementary Figure 20.** Summary receiver operating characteristic curve for the diagnostic performance of radiomics-based ai models on external validation sets in predicting major pathological response.	42](#_Toc235031329)

[**Supplementary Figure 21.** Summary receiver operating characteristic curve of computed tomography-based radiomics models for predicting major pathological response.	43](#_Toc235031330)

[**Supplementary Figure 22.** Forest plot of the sensitivity of positron emission tomography/computed tomography-based radiomics for predicting major pathological response.	44](#_Toc235031331)

[**Supplementary Figure 23.** Forest plot of the specificity of positron emission tomography/computed tomography-based radiomics for predicting major pathological response.	45](#_Toc235031332)

### **Supplementary Table 1.** Search strategy in PubMed, Embase, Cochrane and Web of Science.

| **Database** | **Search strategy** | **Filters and Limits** | **Number of Studies** |
| --- | --- | --- | --- |
| PubMed | ("Radiomics"[Mesh] OR "Artificial Intelligence"[Mesh] OR "Machine Learning"[Mesh] OR "Deep Learning"[Mesh] OR "Artificial Intelligence"[Title/Abstract] OR "AI"[Title/Abstract] OR "Machine Learning"[Title/Abstract] OR "Deep Learning"[Title/Abstract] OR "Machine Intelligence"[Title/Abstract] OR "Radiomic"[Title/Abstract] OR "convolutional neural network"[Title/Abstract] OR "Transfer Learning"[Title/Abstract] OR "Ensemble Learning"[Title/Abstract] OR "Prediction model"[Title/Abstract] OR "Prediction models"[Title/Abstract] OR "random forest"[Title/Abstract] OR "neural network"[Title/Abstract] OR "neural networks"[Title/Abstract] OR "CNN"[Title/Abstract] OR "Support vector machine"[Title/Abstract] OR "SVM"[Title/Abstract] OR "Gradient Boosting Machine"[Title/Abstract] OR "GBM"[Title/Abstract] OR "Nomogram"[Title/Abstract] OR "XGBoost"[Title/Abstract] OR "Adaboost"[Title/Abstract] OR "Decision tree"[Title/Abstract] OR "ResNet"[Title/Abstract] OR "VGG"[Title/Abstract] OR "Naive Bayesian"[Title/Abstract] OR "Multilayer perceptron"[Title/Abstract] OR "Bayesian network"[Title/Abstract]) AND ("Carcinoma, Non-Small-Cell Lung"[Mesh] OR "non-small cell lung cancer"[Title/Abstract] OR "non small cell lung cancer"[Title/Abstract] OR “NSCLC”[Title/Abstract] OR "lung adenocarcinoma"[Title/Abstract] OR "lung squamous cell carcinoma"[Title/Abstract] OR "large cell carcinoma of lung"[Title/Abstract]) AND (“Neoadjuvant Therapy”[Mesh] OR "neoadjuvant immunochemotherapy"[Title/Abstract] OR "neoadjuvant immuno-chemotherapy"[Title/Abstract] OR "neoadjuvant immunotherapy"[Title/Abstract] OR "neoadjuvant immune checkpoint inhibitor*"[Title/Abstract] OR "neoadjuvant"[Title/Abstract] OR “chemoimmunotherapy”[Title/Abstract] OR “NACI”[Title/Abstract] OR “immunochemotherapy”[Title/Abstract]) | No restrictions applied regarding date range, language, or publication type. | 130 |
| Embase | ('radiomics'/exp OR 'artificial intelligence'/exp OR 'machine learning'/exp OR 'deep learning'/exp OR 'artificial intelligence':ab,ti OR 'ai':ab,ti OR 'machine learning':ab,ti OR 'deep learning':ab,ti OR 'machine intelligence':ab,ti OR 'radiomic':ab,ti OR 'convolutional neural network':ab,ti OR 'transfer learning':ab,ti OR 'ensemble learning':ab,ti OR 'prediction model':ab,ti OR 'prediction models':ab,ti OR 'random forest':ab,ti OR 'neural network':ab,ti OR 'neural networks':ab,ti OR 'cnn':ab,ti OR 'support vector machine':ab,ti OR 'svm':ab,ti OR 'gradient boosting machine':ab,ti OR 'gbm':ab,ti OR 'nomogram':ab,ti OR 'xgboost':ab,ti OR 'adaboost':ab,ti OR 'decision tree':ab,ti OR 'resnet':ab,ti OR 'vgg':ab,ti OR 'naive bayesian':ab,ti OR 'multilayer perceptron':ab,ti OR 'bayesian network':ab,ti) AND ('non small cell lung cancer'/exp OR 'non-small cell lung cancer':ab,ti OR 'non small cell lung cancer':ab,ti OR 'nsclc':ab,ti OR 'lung adenocarcinoma':ab,ti OR 'lung squamous cell carcinoma':ab,ti OR 'large cell carcinoma of lung':ab,ti) AND ('neoadjuvant therapy'/exp OR 'chemoimmunotherapy'/exp OR 'neoadjuvant immunochemotherapy':ab,ti OR 'neoadjuvant immuno-chemotherapy':ab,ti OR 'neoadjuvant immunotherapy':ab,ti OR 'neoadjuvant immune checkpoint inhibitor*':ab,ti OR 'neoadjuvant':ab,ti OR 'chemoimmunotherapy':ab,ti OR 'naci':ab,ti OR 'immunochemotherapy':ab,ti) | No restrictions applied regarding date range, language, or publication type. | 383 |
| Web of Science | TS=("Radiomics" OR "Artificial Intelligence" OR "AI" OR "Machine Learning" OR "Deep Learning" OR "Machine Intelligence" OR "Radiomic" OR "convolutional neural network" OR "Transfer Learning" OR "Ensemble Learning" OR "Prediction model" OR "random forest" OR "neural network" OR "CNN" OR "Support vector machine" OR "SVM" OR "Gradient Boosting Machine" OR "GBM" OR "Nomogram" OR "XGBoost" OR "Adaboost" OR "Decision tree" OR "ResNet" OR "VGG" OR "Naive Bayesian" OR "Multilayer perceptron" OR "Bayesian network")  AND TS=("non-small cell lung cancer" OR "NSCLC" OR "lung adenocarcinoma" OR "lung squamous cell carcinoma" OR "large cell carcinoma of lung") AND TS=("neoadjuvant therapy" OR "neoadjuvant immunochemotherapy" OR "neoadjuvant immunotherapy" OR "neoadjuvant immune checkpoint inhibitor*" OR "neoadjuvant" OR "chemoimmunotherapy" OR "NACI" OR "immunochemotherapy") | No restrictions applied regarding date range, language, or publication type. | 151 |
| Cochrane Library | ("Radiomics" OR "Artificial Intelligence" OR "AI" OR "Machine Learning" OR "Deep Learning" OR "Machine Intelligence" OR "Radiomic" OR "convolutional neural network" OR "Transfer Learning" OR "Ensemble Learning" OR "Prediction model" OR "random forest" OR "neural network" OR "CNN" OR "Support vector machine" OR "SVM" OR "Gradient Boosting Machine" OR "GBM" OR "Nomogram" OR "XGBoost" OR "Adaboost" OR "Decision tree" OR "ResNet" OR "VGG" OR "Naive Bayesian" OR "Multilayer perceptron" OR "Bayesian network") AND ("non-small cell lung cancer" OR "NSCLC" OR "lung adenocarcinoma" OR "lung squamous cell carcinoma" OR "large cell carcinoma of lung") AND ("neoadjuvant therapy" OR "neoadjuvant immunochemotherapy" OR "neoadjuvant immunotherapy" OR "neoadjuvant immune checkpoint inhibitor" OR "neoadjuvant" OR "chemoimmunotherapy" OR "NACI" OR "immunochemotherapy") | No restrictions applied regarding date range, language, or publication type. | 33 |

### **Supplementary Table 2.** Risk of bias assessment (PROBAST+AI) model development based on seven domains.

| **Author, year** | | **Quality** | | | | **Applicability concerns** | | | **Overall judgement** | |
| --- | --- | --- | --- | --- | --- | --- | --- | --- | --- | --- |
|  |  | **Participants and data sources ^a^** | **Predictors ^b^** | **Outcome ^c^** | **Analysis ^d^** | **Participants and data sources ^e^** | **Predictors ^f^** | **Outcome ^g^** | **Quality ^h^** | **Applicability concerns ^i^** |
| Deng et al. | 2025 | L | L | L | H | L | L | L | H | L |
| Yang et al. | 2025 | L | L | L | L | L | L | L | L | L |
| Bao et al. | 2025 | L | L | L | L | L | L | L | L | L |
| Liu et al. | 2024 | L | L | L | L | L | L | L | L | L |
| Liu et al. | 2025 | U | L | L | L | L | L | L | U | L |
| Yang et al. | 2023 | L | L | L | L | L | L | L | L | L |
| Qu et al. | 2024 | L | L | L | L | L | L | L | L | L |
| Ye et al. | 2024 | L | L | L | L | L | L | L | L | L |
| Fan et al. | 2025 | L | L | L | L | L | L | L | L | L |
| Ma et al. | 2025 | L | U | L | L | L | L | L | U | L |
| Zheng et al. | 2025 | L | L | L | L | L | L | L | L | L |
| Han et al. | 2025 | L | L | L | L | L | L | L | L | L |
| Liu et al. | 2023 | H | L | L | L | L | L | L | H | L |
| Geng et al. | 2025 | L | L | L | L | L | L | L | L | L |
| Zhang et al. | 2025 | L | L | L | L | L | L | L | L | L |
| Sheng et al. | 2025 | L | L | L | L | L | L | L | L | L |
| Han et al. | 2024 | U | L | L | L | L | L | L | U | L |
| Peng et al. | 2025 | L | L | L | L | L | L | L | L | L |
| Ye et al. | 2024 | L | L | L | L | L | L | L | L | L |
| Gan et al. | 2025 | L | L | L | L | L | L | L | L | L |
| Ye et al. | 2025 | L | L | L | L | L | L | L | L | L |
| Yang et al. | 2025 | L | L | U | L | L | L | L | U | L |
| Yang et al. | 2024 | L | L | L | L | L | L | L | L | L |

**Abbreviation:** PROBAST+AI, Prediction model Risk of Bias Assessment Tool + AI, L low; H high; U unclear.

**Footnote:** Signaling questions are rated as "yes" (Y), "probably yes" (PY), "probably no" (PN), "no" (N), "no information" (NI), and in some cases "not applicable" (NA). All signaling questions are phrased in such a way that "yes" or "probably yes" indicates a low risk of bias. Any signaling questions rated as "no" or "probably no" indicate a potential high risk of bias in that domain. If there are no "no" or "probably no" ratings, but "no information" (NI) is present, the risk of bias in that domain is classified as unclear.

**a. Participants and data sources**

1.1 Were appropriate data sources used?

1.2 Was an appropriate study design used?

1.3 Did the in- and exclusions of study participants result in a representative dataset?

**b. Predictors**

2.1 Were predictors defined and assessed in a similar way for all participants?

2.2 Was any pre-processing of predictors similar for all participants?

2.3 Were predictor assessments made without knowledge of outcome data?

2.4 Were the predictors included in the model available at the time the model was intended to be used?

**c. Outcome**

3.1 Were outcomes defined and assessed appropriately?

3.2 Were outcomes defined and assessed in a similar way for all participants?

3.3 Were outcome assessments made without use or knowledge of predictor data?

3.4 Was the time interval between predictor assessment and outcome assessment appropriate?

**d. Analysis**

4.1 Was there evidence that the sample size was reasonable?

**e. Participants and data sources**

Concern that the (data of the) included participants do not match the review question or the assessor’s intended use of the prediction model.

f. **Predictors**

Concern that the definition, pre-processing, assessment, or timing of assessment of the predictors in the model do not match the review question or the assessor’s intended use.

g. **Outcome**

Concern that the outcome, its definition, assessment, or timing of assessment do not match the review question or the assessor’s intended use.

**h. Quality**

Low risk: If all four domains were rated low concern regarding quality.

High risk: If at least one domain was rated high concern regarding quality .

Unclear: If at least one domain was rated unclear concern regarding quality and no domains were rated high concern.

**i. Applicability concerns**

Low risk: If all three domains were rated low concern for applicability.

High risk: If at least one domain was rated high concern for applicability.

Unclear: If at least one domain was rated unclear concern for applicability and no domains were rated high concern.

### **Supplementary Table 3.** Risk of bias assessment (PROBAST+AI) model evaluation based on seven domains.

| **Author, year** | | **Risk of bias** | | | | | | | | **Applicability concerns** | | | | | | **Overall judgement** | |
| --- | --- | --- | --- | --- | --- | --- | --- | --- | --- | --- | --- | --- | --- | --- | --- | --- | --- |
|  |  | **Participants and data sources ^a^** | | **Predictors ^b^** | | **Outcome ^c^** | | **Analysis ^d^** | | **Participants and data sources ^e^** | | **Predictors ^f^** | | **Outcome ^g^** | | **Risk of bias ^h^** | **Applicability concerns ^i^** |
| Deng et al. | 2025 | | L | | L | | L | | H | | L | | L | | L | H | L |
| Yang et al. | 2025 | | L | | L | | L | | L | | L | | L | | L | L | L |
| Bao et al. | 2025 | | L | | L | | L | | L | | L | | L | | L | L | L |
| Liu et al. | 2024 | | L | | L | | L | | H | | L | | L | | L | H | L |
| Liu et al. | 2025 | | U | | L | | L | | L | | L | | L | | L | U | L |
| Yang et al. | 2023 | | L | | L | | L | | H | | L | | L | | L | H | L |
| Qu et al. | 2024 | | L | | L | | L | | L | | L | | L | | L | L | L |
| Ye et al. | 2024 | | L | | L | | L | | L | | L | | L | | L | L | L |
| Fan et al. | 2025 | | L | | L | | L | | L | | L | | L | | L | L | L |
| Ma et al. | 2025 | | L | | U | | L | | H | | L | | L | | L | H | L |
| Zheng et al. | 2025 | | L | | L | | L | | L | | L | | L | | L | L | L |
| Han et al. | 2025 | | L | | L | | L | | L | | L | | L | | L | L | L |
| Liu et al. | 2023 | | H | | L | | L | | H | | L | | L | | L | H | L |
| Geng et al. | 2025 | | L | | L | | L | | L | | L | | L | | L | L | L |
| Zhang et al. | 2025 | | L | | L | | L | | L | | L | | L | | L | L | L |
| Sheng et al. | 2025 | | L | | L | | L | | L | | L | | L | | L | L | L |
| Han et al. | 2024 | | U | | L | | L | | H | | L | | L | | L | H | L |
| Peng et al. | 2025 | | L | | L | | L | | L | | L | | L | | L | L | L |
| Ye et al. | 2024 | | L | | L | | L | | L | | L | | L | | L | L | L |
| Gan et al. | 2025 | | L | | L | | L | | L | | L | | L | | L | L | L |
| Ye et al. | 2025 | | L | | L | | L | | L | | L | | L | | L | L | L |
| Yang et al. | 2025 | | L | | L | | U | | H | | L | | L | | L | H | L |
| Yang et al. | 2024 | | L | | L | | L | | L | | L | | L | | L | L | L |

**Abbreviation:** PROBAST+AI, Prediction model Risk of Bias Assessment Tool + AI, L low; H high; U unclear.

**Footnote:** Signaling questions are rated as "yes" (Y), "probably yes" (PY), "probably no" (PN), "no" (N), "no information" (NI), and in some cases "not applicable" (NA). All signaling questions are phrased in such a way that "yes" or "probably yes" indicates a low risk of bias. Any signaling questions rated as "no" or "probably no" indicate a potential high risk of bias in that domain. If there are no "no" or "probably no" ratings, but "no information" (NI) is present, the risk of bias in that domain is classified as unclear.

**a. Participants and data sources**

1.1 Were appropriate data sources used?

1.2 Was an appropriate study design used?

1.3 Did the in- and exclusions of study participants result in a representative dataset?

**b. Predictors**

2.1 Were predictors defined and assessed in a similar way for all participants?

2.2 Was any pre-processing of predictors similar for all participants?

2.3 Were predictor assessments made without knowledge of outcome data?

2.4 Were the predictors included in the model available at the time the model was intended to be used?

**c. Outcome**

3.1 Were outcomes defined and assessed appropriately?

3.2 Were outcomes defined and assessed in a similar way for all participants?

3.3 Were outcome assessments made without use or knowledge of predictor data?

3.4 Was the time interval between predictor assessment and outcome assessment appropriate?

**d. Analysis**

4.1 Was there evidence that the sample size was reasonable?

**e. Participants and data sources**

Concern that the (data of the) included participants do not match the review question or the assessor’s intended use of the prediction model.

f. **Predictors**

Concern that the definition, pre-processing, assessment, or timing of assessment of the predictors in the model do not match the review question or the assessor’s intended use.

g. **Outcome**

Concern that the outcome, its definition, assessment, or timing of assessment do not match the review question or the assessor’s intended use.

**h. Risk of bias**

Low risk: If all four domains were rated low risk of bias.

High risk: If at least one domain was rated high risk of bias.

Unclear: If at least one domain was rated unclear risk of bias and no domains were rated high risk of bias.

**i. Applicability concerns**

Low risk: If all three domains were rated low concern for applicability.

High risk: If at least one domain was rated high concern for applicability.

Unclear: If at least one domain was rated unclear concern for applicability and no domains were rated high concern.

### **Supplementary Table 4**. Grading of recommendations, assessment, development, and evaluations (GRADE) scoring assessments in all the pooled outcomes.

| **Dataset** | **Outcome** | **Risk of Bias ^a^** | **Inconsistency ^b^** | **Indirectness ^c^** | **Imprecision ^d^** | **Publication Bias ^e^** | **Total Downgrade** | **Final Rating** |
| --- | --- | --- | --- | --- | --- | --- | --- | --- |
| Radiomics-based AI for pCR prediction | Sensitivity | 1 | 0 | 0 | 1 | 0 | 2 | Low |
|  | Specificity | 1 | 0 | 0 | 1 | 0 | 2 | Low |
|  | AUC | 1 | 0 | 0 | 0 | 0 | 1 | Moderate |
| Radiomics-based AI for MPR prediction | Sensitivity | 1 | 0 | 0 | 1 | 0 | 2 | Low |
|  | Specificity | 1 | 0 | 0 | 1 | 0 | 2 | Low |
|  | AUC | 1 | 0 | 0 | 0 | 0 | 1 | Moderate |

AUC area under the curve; pCR pathological complete response; MPR major pathological response

a. Risk of Bias

Assessed using tools (PROBAST+AI) to evaluate study design and methodology.

Downgrade by 1 level if at least one study has a high risk of bias.

b. Inconsistency

Measured using I² statistics to assess heterogeneity across studies.

Downgrade by 1 level if I² > 50% and the source of heterogeneity cannot be explained.

If the heterogeneity is identified (e.g., through meta-regression or bivariate box plot), no downgrade is applied.

c. Indirectness

Evaluates whether study populations, interventions, or outcomes differ from the target research question.

Outcome Indirectness: Downgrade if inconsistent outcome measures are used, such as estimating sensitivity/specificity indirectly from ROC-based Youden Index.

d. Imprecision

Assessed based on confidence intervals (CIs).

Downgrade if 95% CI width > 0.20 or if CIs cross clinical thresholds (e.g., 0.80).

e. Publication Bias

Evaluated using Deek’s Funnel Plot or similar tools.

Downgrade by 1 level if the funnel plot shows significant asymmetry or if *P*-value < 0.10 in Deek’s tests.

### **Supplementary Table 5.** Technical aspects information and diagnostic raw data of included studies for pCR prediction.

| **Author** | **Year** | **Type of imaging** | **Data source** | **AI model** | **Data splitting method** | **AI method** | **AI algorithms^a^** | **Internal validation** | | | | | **External validation** | | | | |
| --- | --- | --- | --- | --- | --- | --- | --- | --- | --- | --- | --- | --- | --- | --- | --- | --- | --- |
|  |  |  |  |  |  |  |  | **TP** | **FP** | **FN** | **TN** | **AUC** | **TP** | **FP** | **FN** | **TN** | **AUC** |
| Deng et al. | 2025 | PET/CT | Local hospital | Radiomics & clinical model | Independent dataset validation | ML | LR | NA | NA | NA | NA | NA | 3 | 0 | 0 | 5 | NA |
| Yang et al. | 2025 | CT | Local hospital | Radiomics model | Hold-out | ML | LDA | 29 | 11 | 11 | 41 | 0.827 | NA | NA | NA | NA | NA |
| Liu et al. | 2024 | PET/CT | Local hospital | Radiomics & clinical model | Hold-out | ML | SVM | 14 | 3 | 0 | 15 | 0.857 | NA | NA | NA | NA | NA |
| Liu et al. | 2025 | PET/CT | Local hospital | Radiomics & clinical model | Hold-out | ML | RF | 14 | 6 | 7 | 36 | 0.823 | NA | NA | NA | NA | NA |
| Yang et al. | 2023 | CT | Local hospital | Radiomics & clinical model | Hold-out | ML | LR | 15 | 3 | 3 | 12 | 0.857 | NA | NA | NA | NA | NA |
| Qu et al. | 2024 | CT | Local hospital | Radiomics & clinical model | Hold-out | DL | CNN-  ResNet-152 | 18 | 12 | 6 | 33 | 0.775 | 19 | 22 | 5 | 29 | 0.640 |
| Ye et al. | 2024 | CT | Local hospital | Radiomics & clinical model | Hold-out | ML | RF | 8 | 5 | 0 | 9 | 0.884 | 37 | 19 | 3 | 53 | 0.866 |
| Fan et al. | 2025 | CT | Local hospital | Radiomics model | Hold-out | ML | LR | 19 | 11 | 14 | 21 | 0.752 | NA | NA | NA | NA | NA |
| Sheng et al. | 2025 | PET/CT | Local hospital | Radiomics model | Hold-out | ML | GBM | 18 | 7 | 8 | 28 | 0.828 | NA | NA | NA | NA | NA |
| Ye et al. | 2024 | CT | Local hospital | Radiomics & clinical model | Independent dataset validation | ML | LR | NA | NA | NA | NA | NA | 18 | 13 | 5 | 34 | 0.781 |
| Ye et al. | 2025 | CT | Local hospital | Radiomics & clinical model | Hold-out | ML | XGBClassifier | 25 | 3 | 5 | 45 | 0.889 | 28 | 7 | 24 | 89 | 0.870 |
| Yang et al. | 2025 | CT | Local hospital | Radiomics model | Hold-out | DL | A2ST-GCM | 6 | 1 | 1 | 11 | 0.886 | NA | NA | NA | NA | NA |
| Yang et al. | 2024 | PET/CT | Local hospital | Radiomic model | Independent dataset validation | ML | LASSO | NA | NA | NA | NA | NA | 22 | 12 | 6 | 44 | 0.818 |

ML machine learning; DL deep learning; TP true positive; TN true negative; FP false positive; FN false positive; NA not available; LR logistic regression; LDA linear discriminant analysis; SVM support vector machine; RF random forest; CNN convolutional neural network; A2ST-GCM adaptive spatio-temporal graph convolutional model; LASSO least absolute shrinkage and selection operator.

^a^ The optimal means the highest AUC value in the included study.

### **Supplementary Table 6.** Technical aspects information and diagnostic raw data of included studies for MPR prediction.

| **Author** | **Year** | **Type of imaging** | **Data source** | **AI model** | **Data splitting method** | **AI method** | **AI algorithms ^a^** | **Internal validation** | | | | | **External validation** | | | | |
| --- | --- | --- | --- | --- | --- | --- | --- | --- | --- | --- | --- | --- | --- | --- | --- | --- | --- |
|  |  |  |  |  |  |  |  | **TP** | **FP** | **FN** | **TN** | **AUC** | **TP** | **FP** | **FN** | **TN** | **AUC** |
| Bao et al. | 2025 | CT | Local hospital | Radiomics model | Hold-out | ML | LDA | NA | NA | NA | NA | NA | 31 | 9 | 25 | 21 | 0.69 |
| Ma et al. | 2025 | PET/CT | Local hospital | Radiomics & clinical model | Hold-out | ML | LR | 13 | 0 | 8 | 12 | 0.78 | NA | NA | NA | NA | NA |
| Zheng et al. | 2025 | CT | Local hospital | Radiomics & clinical model | Independent dataset validation | DL | CNN-MobileNet | 18 | 7 | 8 | 17 | 0.760 | 21 | 8 | 9 | 21 | 0.787 |
| Han et al. | 2025 | CT | Local hospital | Radiomics & clinical model | Hold-out | ML | XGBoost | 39 | 9 | 4 | 9 | 0.831 | 21 | 6 | 2 | 7 | 0.799 |
| Liu et al. | 2023 | CT | Local hospital | Radiomics & clinical model | Hold-out | ML | LR | 12 | 1 | 4 | 8 | 0.80 | NA | NA | NA | NA | NA |
| Geng et al. | 2025 | CT | Local hospital | Radiomics model | Hold-out | DL | CNN-GoogLeNet | 56 | 4 | 10 | 46 | 0.973 | 20 | 2 | 2 | 20 | 0.942 |
| Zhang et al. | 2025 | PET/CT | Local hospital | Radiomics & clinical model | Independent dataset validation | ML | LASSO | NA | NA | NA | NA | NA | 28 | 4 | 2 | 51 | 0.809 |
| Han et al. | 2024 | CT | Local hospital | Radiomics & clinical model | Hold-out | ML | LR | 13 | 6 | 13 | 18 | 0.761 | NA | NA | NA | NA | NA |
| Peng et al. | 2025 | CT | Local hospital | Radiomics & clinical model | Independent dataset validation | DL | CNN-ResNet-50 | NA | NA | NA | NA | NA | 28 | 0 | 4 | 28 | 0.950 |
| Gan et al. | 2025 | CT | Local hospital | Radiomics & clinical model | Hold-out | DL | Transformer | 13 | 2 | 4 | 16 | 0.923 | 84 | 18 | 10 | 41 | 0.858 |

ML machine learning; DL deep learning; TP true positive; TN true negative; FP false positive; FN false positive; NA not available; LR logistic regression; LDA linear discriminant analysis; CNN convolutional neural network; LASSO least absolute shrinkage and selection operator

^a^ The optimal means the highest AUC value in the included study.

### **Supplementary Table 7.** Diagnostic data for different models.

| **Author** | **Year** | **Target condition** | **PERCIST** | | | | **RECIST 1.1** | | | |
| --- | --- | --- | --- | --- | --- | --- | --- | --- | --- | --- |
|  |  |  | **TP** | **FP** | **FN** | **TN** | **TP** | **FP** | **FN** | **TN** |
| Deng et al. | 2025 | pCR | 14 | 1 | 5 | 16 | 2 | 0 | 17 | 17 |
| Bao et al. (internal) | 2025 | MPR | NA | NA | NA | NA | 22 | 9 | 20 | 29 |
| Bao et al.(external) | 2025 | MPR | NA | NA | NA | NA | 12 | 4 | 44 | 26 |
| Han et al. (internal) | 2024 | MPR | NA | NA | NA | NA | 20 | 15 | 6 | 9 |

MPR major pathological response; pCR pathological complete response; TP true positive; TN true negative; FP false positive; FN false negative; NA not available.

### **Supplementary Table 8.** Technical aspects of included studies for CT.

| **Author** | **Year** | **Scanner Modality (Manufacturer)** | **Evaluation Time** | **Regions of interest** | **Enhancement phase** | **Contrast-enhanced** |
| --- | --- | --- | --- | --- | --- | --- |
| Yang et al. | 2025 | Discovery CT750 HD (GE Medical Systems), Optima 680 Expert (GE Medical Systems), Revolution EVO (GE Medical Systems), SOMATOM Definition AS+ (Siemens), SOMATOM Drive (Siemens), IQon spectral CT (Philips) | Pre-treatment | Semi-automatic | NA | NO |
| Bao et al. | 2025 | NA | Pre-treatment & post-treatment | Manually | venous phase | Yes |
| Yang et al. | 2023 | NA | Pre-treatment | Manually | NA | NO |
| Qu et al. | 2024 | Somatom Deﬁnition AS+ (Siemens Medical Systems), iCT256 (Philips Medical Systems) | Pre-treatment | Semi-automatic | NA | NO |
| Ye et al. | 2024 | NA | Pre-treatment | Manually | NA | YES |
| Fan et al. | 2025 | Somatom Sensation 64 (Siemens Medical Solutions), Lightspeed 16 (GE Medical Systems), Discovery CT 750 HD (GE Medical Systems) | Pre-treatment | Semi-automatic | NA | NO |
| Zheng et al. | 2025 | NA | Pre-treatment | Manually | NA | NO |
| Han et al. | 2025 | Philips Brilliance models 128 (Philips), Philips Brilliance models 256 (Philips), Siemens Somatom Definition AS 128 (Siemens Medical Systems), Philips IQon Spectral CT (Philips), GE Revolution 256 CT (GE Medical Systems) | Pre-treatment | Semi-automatic | NA | NO |
| Liu et al. | 2023 | Somatom Deﬁnition Flash (Siemens), uCT780 (United Imaging), Somatom Perspective 128(Siemens), Somatom (Siemens) | Pre-treatment | Manually | NA | YES |
| Geng et al. | 2025 | NA | Pre-treatment | Manually | NA | YES |
| Han et al. | 2024 | SOMATOM Definition AS + (Siemens), SOMATOM Definition Flash (Siemens), LightSpeed (GE) | Pre-treatment & post-treatment | Manually | NA | YES |
| Peng et al. | 2025 | NA | Pre-treatment | Semi-automatic | NA | YES |
| Ye et al. | 2024 | Siemens, GE | Pre-treatment | Manually | arterial phase | YES |
| Gan et al. | 2025 | Somatom Definition AS+ (Siemens), SOMATOM Force (Siemens), SOMATOM Definition Flash (Siemens), Brilliance 40 (Philips), iCT 256 (Philips), Ingenuity Flex 32 (Philips), IQon Spectral CT (Philips), Discovery CT750 HD (GE), uCT 510 (United Imaging), uCT 780 (United Imaging) | Pre-treatment | Manually | NA | YES |
| Ye et al. | 2025 | Siemens, GE | Pre-treatment | Semi-automatic | NA | YES |
| Yang et al. | 2025 | NA | Pre-treatment & post-treatment | Manually | NA | NO |

NA, not available; CT, computed tomography.

### **Supplementary Table 9.** Technical aspects of included studies for PET/CT.

| **Author** | **Year** | **Scanner Modality (Manufacturer)** | **Evaluation Time** | **Regions of interest** | **Radiotracers** | **Time for injection** | **Dose** |
| --- | --- | --- | --- | --- | --- | --- | --- |
| Deng et al. | 2025 | Discovery VCT (GE Healthcare) | During treatment | Semi-automatic | [^18^F]FDG | 60 minutes uptake time | 3.7 to 5 MBq/kg |
| Liu et al. | 2024 | GE Discovery Elite PET/CT scanner (GE Medical Systems) | Pre-treatment | Semi-automatic | [^18^F] FDG | 60 minutes uptake time | 3.7 to 4.44 MBq/kg |
| Liu et al. | 2025 | GE Discovery Elite PET/CT scanner (GE Medical Systems) | Pre-treatment | Semi-automatic | [^18^F] FDG | 60 minutes uptake time | 4.2 MBq/kg |
| Ma et al. | 2025 | Discovery 690 Elite (GE Healthcare) | Pre-treatment | Semi-automatic | [^18^F] FDG | 60 minutes uptake time | 3.70–5.55 MBq/kg |
| Zhang et al. | 2025 | PET VCAR software (GE Healthcare) | Pre-treatment | Manually | [^18^F] FDG | 60 minutes uptake time | 3.7–7.4 MBq/kg |
| Sheng et al. | 2025 | GE Millennium VG (GE Healthcare) | Pre-treatment & post-treatment | Manually | [^18^F] FDG | 60 minutes uptake time | 3.08–6.71 MBq/kg |
| Yang et al. | 2024 | Siemens Bio-graph 64 (Siemens Medical Systems), General Electric Discovery 710 (GE Healthcare), General Electric Discovery STE (GE Healthcare) | Pre-treatment | Manually | [^18^F] FDG | 60 minutes uptake time | 3.7–5.6 MBq/kg |

PET/CT, positron emission tomography - computed tomography.

### **Supplementary Table 10.** AI reporting transparency assessment across included studies

| **Study** | **Calibration reported** | **External validation** | **Explainability methods** | **Code / Model available** | **TRIPOD-AI / CONSORT-AI** |
| --- | --- | --- | --- | --- | --- |
| Bao et al., 2025 | Yes (calibration plot) | External | Yes (SHAP) | Yes (mentioned) | No |
| Deng et al., 2025 | No | External | Yes (SHAP) | No | No |
| Fan et al., 2025 | No | Internal | Yes (SHAP) | No | No |
| Gan et al., 2025 | Yes (calibration plot) | External | Yes (attention map, interpretability) | Yes (github.com/onekeyai-platform) | No |
| Geng et al., 2025 | Yes (calibration plot) | External | Yes (SHAP, Grad-CAM, attention map) | Yes (github.com/onekeyai-platform/onekey) | No |
| Han et al., 2024 | Yes (calibration plot) | Internal | Yes (SHAP) | No | No |
| Han et al., 2025 | Yes (calibration plot) | External | Yes (SHAP, Grad-CAM) | No | Yes (TRIPOD) |
| Liu et al., 2023 | Yes (calibration plot, Hosmer-Lemeshow) | Internal | Yes (SHAP) | No | No |
| Liu et al., 2024 | Yes (calibration plot) | Internal | Yes (SHAP) | No | No |
| Liu et al., 2025 | Yes (calibration plot) | Internal | Yes (SHAP) | No | No |
| Ma et al., 2025 | Yes (calibration plot) | Internal | No | No | No |
| Peng et al., 2025 | No | External | Yes (Grad-CAM, interpretability) | No | No |
| Qu et al., 2024 | No | External | Yes (SHAP) | No | No |
| Sheng et al., 2025 | Yes (calibration plot) | Internal | Yes (SHAP, feature importance) | No | Yes (TRIPOD) |
| Yang (A2ST-GCM) et al., 2025 | No | Internal | Yes (SHAP, Grad-CAM, saliency map) | No | No |
| Yang (Rim) et al., 2025 | Yes (calibration plot, Hosmer-Lemeshow) | Internal | Yes (SHAP) | No | Yes (TRIPOD) |
| Yang et al., 2023 | No | Internal | Yes (SHAP) | No | No |
| Yang et al., 2024 | Yes (calibration plot) | Internal | No | No | No |
| Ye (CT) et al., 2024 | Yes (calibration plot) | External | Yes (SHAP) | No | Yes (TRIPOD) |
| Ye (Multimodal) et al., 2024 | No | External | Yes (SHAP, saliency map, feature importance) | No | Yes (TRIPOD) |
| Ye et al., 2025 | No | External | Yes (SHAP) | Yes (github.com/zhenweishi/lc-nicer) | Yes (TRIPOD) |
| Zhang et al., 2025 | Yes (calibration plot) | Internal | No | No | No |
| Zheng et al., 2025 | No | External | No | No | No |

TRIPOD-AI, Transparent Reporting of a Multivariable Prediction Model for Individual Prognosis or Diagnosis – Artificial Intelligence; CONSORT-AI, Consolidated Standards of Reporting Trials – Artificial Intelligence; SHAP, SHapley Additive exPlanations; Grad-CAM, Gradient-weighted Class Activation Mapping; NA, Not applicable / Not reported.

### **Supplementary Table 11**. Subgroup analysis for performance comparison for pCR.

| **Subgroup** | **Sample size** | **Number of validation data sets, n** | **Sensitivity(95%C****I)** | **Subgroup difference ^a^** | **Specificity(95%CI)** | **Subgroup difference ^a^** | **AUC (95%CI)** | **Subgroup difference ^a^** |
| --- | --- | --- | --- | --- | --- | --- | --- | --- |
| **AI versus traditional model** |  |  |  |  |  |  |  |  |
| Radiomics-based AI | 1031 | 16 | 0.77 (0.70-0.83) | Z = 3.97, *P* < 0.001 | 0.79 (0.73-0.84) | Z = 3.79, *P* < 0.001 | 0.85 (0.82- 0.88) | NA |
| Radiological response criteria | 72 | 2 | 0.42 (0.26-0.59) |  | 0.97 (0.85-1.00) |  | NA |  |
| **Validation type** |  |  |  |  |  |  |  |  |
| AI in internal validation | 618 | 10 | 0.76(0.68 - 0.85) | Z = 0.44, *P* = 0.66 | 0.80(0.74 - 0.87) | Z = 0.35, *P* = 0.72 | 0.85 (0.82- 0.88) | Z = 0.00, *P* = 1.00 |
| AI in external validation | 413 | 6 | 0.79(0.68 - 0.89) |  | 0.78(0.69 - 0.87) |  | 0.85 (0.82- 0.88) |  |
| **Imaging type** |  |  |  |  |  |  |  |  |
| CT-based AI | 783 | 11 | 0.77(0.69 - 0.84) | Z = 0.28, *P* = 0.78 | 0.78(0.72 - 0.84) | Z= 0.91, *P* = 0.36 | 0.84 (0.81- 0.87) | Z = 0.46, *P* = 0.64 |
| PET/CT-based AI | 248 | 5 | 0.79(0.67 - 0.91) |  | 0.83(0.74 - 0.92) |  | 0.83 (0.80- 0.86) |  |

pCR pathological complete response; AUC area under the curve, NA not available.

**^a^** Based on two-sided Z-test.

### **Supplementary Table 12.** Subgroup analysis for performance comparison for MPR.

| **Subgroup** | **Sample size** | **Number of validation data sets, n** | **Sensitivity(95%CI)** | **Subgroup difference ^a^** | **Specificity(95%CI)** | **Subgroup difference ^a^** | **AUC (95%CI)** | **Subgroup difference ^a^** |
| --- | --- | --- | --- | --- | --- | --- | --- | --- |
| **AI versus traditional model** |  |  |  |  |  |  |  |  |
| Radiomics-based AI | 973 | 14 | 0.80 (0.72-0.87) | Z = 6.02, P < 0.001 | 0.83 (0.73-0.90) | Z = 1.94, P = 0.05 | 0.88 (0.85- 0.91) | Z = 3.95, P < 0.001 |
| Radiological response criteria | 216 | 3 | 0.44 (0.35-0.53) |  | 0.70 (0.59-0.79) |  | 0.65 (0.54- 0.76) |  |
| **Validation type** |  |  |  |  |  |  |  |  |
| AI in internal validation | 463 | 7 | 0.75(0.64 - 0.87) | Z = 1.22, P = 0.23 | 0.83 (0.72 - 0.95) | Z = 0.00, P = 1.00 | 0.85 (0.82- 0.88) | Z = 3.01, P = 0.002 |
| AI in external validation | 510 | 7 | 0.84 (0.76 - 0.93) |  | 0.83 (0.72 - 0.94) |  | 0.91 (0.88- 0.93) |  |
| **Imaging type** |  |  |  |  |  |  |  |  |
| CT-based AI | 689 | 12 | 0.80 (0.72 - 0.88) | Z = 0.10, P = 0.92 | 0.80 (0.71 - 0.88) | Z= 2.82, P = 0.005 | 0.87 (0.84- 0.90) | NA |
| PET/CT-based AI | 284 | 2 | 0.81 (0.63 - 1.00) |  | 0.95 (0.88 - 1.00) |  | NA |  |

MPR major pathological response; AUC area under the curve, NA not available.

**^a^** Based on two-sided Z-test.

### **Supplementary Table 13.** Subgroup analysis and meta-regression analysis for pCR**.**

| **Subgroup** | **Number of validation data sets, n** | **Sensitivity(95%CI)** | **Meta-regression *P*-value** | **Specificity(95%CI)** | **Meta-regression *P*-value** |
| --- | --- | --- | --- | --- | --- |
| **AI model** |  |  | 0.01 |  | 0.01 |
| Radiomics model | 5 | 0.71(0.59 - 0.83) |  | 0.78(0.69 - 0.88) |  |
| Radiomics & clinical model | 11 | 0.80(0.72 - 0.87) |  | 0.80(0.73 - 0.86) |  |
| **Data splitting method** |  |  | 0.87 |  | 0.30 |
| Independent dataset validation | 2 | 0.82(0.62 - 1.00) |  | 0.78(0.60 - 0.97) |  |
| Hold-out | 14 | 0.77(0.70 - 0.84) |  | 0.80(0.74 - 0.85) |  |
| **AI method** |  |  | 0.17 |  | 0.41 |
| Machine learning | 13 | 0.77(0.69 - 0.84) |  | 0.81(0.76 - 0.86) |  |
| Deep learning | 3 | 0.79(0.64 - 0.94) |  | 0.70(0.56 - 0.85) |  |
| **Evaluation Time** |  |  | 0.39 |  | 0.66 |
| Pre-treatment & post-treatment | 2 | 0.75(0.53 - 0.97) |  | 0.85(0.70 - 0.99) |  |
| Pre-treatment | 12 | 0.77(0.70 - 0.85) |  | 0.78(0.72 - 0.85) |  |

pCR pathological complete response; AI Artificial intelligence.

### **Supplementary Table 14.** Subgroup analysis and meta-regression analysis for MPR**.**

| **Subgroup** | **Number of validation data sets, n** | **Sensitivity(95%CI)** | **Meta-regression *P*-value** | **Specificity(95%CI)** | **Meta-regression *P*-value** |
| --- | --- | --- | --- | --- | --- |
| **AI model** |  |  | 0.19 |  | 0.76 |
| Radiomics model | 3 | 0.79 (0.63 - 0.94) |  | 0.87 (0.73 - 1.00) |  |
| Radiomics & clinical model | 11 | 0.81 (0.73 - 0.89) |  | 0.82 (0.72 - 0.91) |  |
| **Data splitting method** |  |  | 0.28 |  | 0.77 |
| Independent dataset validation | 4 | 0.82 (0.69 - 0.95) |  | 0.88 (0.77 - 0.99) |  |
| Hold-out | 10 | 0.79 (0.71 - 0.88) |  | 0.81 (0.70 - 0.91) |  |
| **AI method** |  |  | 0.02 |  | 0.06 |
| Machine learning | 7 | 0.77 (0.66 - 0.88) |  | 0.79 (0.66 - 0.92) |  |
| Deep learning | 7 | 0.83 (0.74 - 0.92) |  | 0.86 (0.77 - 0.96) |  |
| **Evaluation Time** |  |  | 0.05 |  | 0.46 |
| Pre-treatment & post-treatment | 1 | 0.55 (0.24 - 0.87) |  | 0.71 (0.31 - 1.00) |  |
| Pre-treatment | 13 | 0.82 (0.75 - 0.88) |  | 0.84 (0.76 - 0.92) |  |

MPR major pathological response; AI Artificial intelligence.

### **Supplementary Figure 1.** Radar plot of the relative diagnostic performance of different radiomics-based artificial intelligence algorithms for predicting pathological complete response.

### **Supplementary Figure 2.** Radar plot of the relative diagnostic performance of different radiomics-based artificial intelligence algorithms for predicting major pathological response.

### **Supplementary Figure 3.** Forest plots of the pooled sensitivity and specificity for predicting pathological complete response based on radiomics-based artificial intelligence.

### **Supplementary Figure 4.** Summary receiver operating characteristic curve for the diagnostic performance of radiomics-based artificial intelligence in predicting pathological complete response.

### **Supplementary Figure 5.** Forest plot of the pooled sensitivity for predicting pathological complete response stratified by RECIST 1.1 and PERCIST models.

### **Supplementary Figure 6.** Forest plot of the pooled specificity for predicting pathological complete response stratified by RECIST 1.1 and PERCIST models.

### **Supplementary Figure 7.** Summary receiver operating characteristic curve for the diagnostic performance of radiomics-based artificial intelligence in predicting pCR after the exclusion of studies with potential patient cohort overlap.

### **Supplementary Figure 8.** Forest plots of the pooled sensitivity and specificity for predicting major pathological response based on radiomics-based artificial intelligence.

### **Supplementary Figure 9.** Summary receiver operating characteristic curve for the diagnostic performance of radiomics-based artificial intelligence in predicting major pathological response.

### **Supplementary Figure 10.** Forest plot of the pooled sensitivity for predicting major pathological response stratified by RECIST 1.1 models.

### **Supplementary Figure 11.** Forest plot of the pooled specificity for predicting major pathological response stratified by RECIST 1.1 model.

### **Supplementary Figure 12.** Summary receiver operating characteristic curve for the diagnostic performance of RECIST 1.1 model in predicting major pathological response.

### **Supplementary Figure 13.** Forest plots of the subgroup analysis for sensitivity and specificity of different artificial intelligence algorithms in predicting pathological complete response.

### **Supplementary Figure 14.** Forest plots of the subgroup analysis for sensitivity and specificity of different artificial intelligence algorithms in predicting major pathological response.

### **Supplementary Figure 15.** Summary receiver operating characteristic curve for the diagnostic performance of radiomics-based ai models on internal validation sets in predicting pathological complete response.

### **Supplementary Figure 16.** Summary receiver operating characteristic curve for the diagnostic performance of radiomics-based ai models on external validation sets in predicting pathological complete response.

### **Supplementary Figure 17.** Summary receiver operating characteristic curve of computed tomography-based radiomics models for predicting pathological complete response.

### **Supplementary Figure 18.** Summary receiver operating characteristic curve of positron emission tomography computed tomography-based radiomics models for predicting pathological complete response.

### **Supplementary Figure 19.** Summary receiver operating characteristic curve for the diagnostic performance of radiomics-based ai models on internal validation sets in predicting major pathological response.

### **Supplementary Figure 20.** Summary receiver operating characteristic curve for the diagnostic performance of radiomics-based ai models on external validation sets in predicting major pathological response.

### **Supplementary Figure 21.** Summary receiver operating characteristic curve of computed tomography-based radiomics models for predicting major pathological response.

### **Supplementary Figure 22.** Forest plot of the sensitivity of positron emission tomography/computed tomography-based radiomics for predicting major pathological response.

### **Supplementary Figure 23.** Forest plot of the specificity of positron emission tomography/computed tomography-based radiomics for predicting major pathological response.
